# Analyzing complex traits and diseases using GxE PRS: genotype-environment interaction in polygenic risk score models

**DOI:** 10.1038/s10038-025-01378-2

**Published:** 2025-08-14

**Authors:** Dovini Jayasinghe, Vu Viet Hoang Pham, Kerri Beckmann, Beben Benyamin, S. Hong Lee

**Affiliations:** 1 Australian Centre for Precision Health, University of South Australia, Adelaide, SA 5000 Australia; 2 UniSA Allied Health and Human Performance, University of South Australia, Adelaide, SA 5000 Australia; 3 South Australian Health and Medical Research Institute (SAHMRI), University of South Australia, Adelaide, SA 5000 Australia; 4 Medicine & Health, University of New South Wales, Sydney, NSW 2052 Australia

**Keywords:** Genetic interaction, Diseases

## Abstract

A deeper understanding of how environmental factors influence genetic risks is crucial for exploring their combined effects on health outcomes. This can be effectively achieved by incorporating genotype-environment (GxE) interactions in polygenic risk score (PRS) models. We applied our recently developed GxEprs model to a wide range of obesity-related complex traits and diseases, leveraging data from the UK Biobank, to capture significant GxE signals. This work represents the first application of the “GxEprs” method, designed to minimize issues with spurious GxE signals and model misspecification. We identified significant GxE signals especially in quantitative phenotypes such as body mass index (BMI), waist-to-hip ratio (WHR), body fat percentage (BF) and waist circumference (WC) and our results indicated a significant enhancement in prediction accuracy in most traits, highlighting the importance of the GxE component. This study demonstrated the potential of incorporating GxE interactions in PRS models, offering a broad understanding of genetic risks and laying foundation in applying these insights in personalized medicine.

## Introduction

Complex traits and diseases, such as body mass index (BMI), waist to hip ratio (WHR), diabetes (DIAB), cardiovascular disorders, and various forms of cancer, are influenced by a complex interplay of genetic and environmental factors [1–6]. By leveraging genomic data, researchers and clinicians can pinpoint genetic variations that contribute significantly to the risk of developing various diseases. This does not only enhance our understanding of the biological mechanisms behind these conditions but also guides the development of targeted therapies and preventive strategies, ultimately improving health outcomes. Better understanding obesity-related traits and diseases is critical due to their widespread impact on global health and their connection to a variety of severe health complications, including DIAB, heart diseases, and certain cancers[7].

Identifying gene-environment (GxE) interactions in genomic prediction is crucial as environmental factors such as diet and physical activity (PA) can significantly modulate an individual’s genetic risk of developing obesity-related conditions[8]. Previous studies [9–11] have reported that the genetic effects of obesity-related traits such as BMI and/or WHR were modulated by alcohol intake and/or PA. Another GxE study reported that genetic effects of hypertension (HYP), an obesity-related disease, were modifying by BMI, WHR and body fat (BF) percentage [12]. Moreover, smoking (SMK) was found to be a modular environmental variable for coronary artery disease (CAD) [13–15].

Genome-wide association studies (GWAS) and genomic prediction offer the potential to identify individuals predisposed to obesity and related comorbidities, facilitating early lifestyle interventions that can alter the progression of disease [16]. Among the genomic prediction tools available, polygenic risk scores (PRSs) have emerged as a significant innovation. PRSs aggregate the effects of numerous genetic variants across the genome to estimate an individual’s genetic liability to develop a disease [17]. For instance, studies have successfully used PRS to predict the risk of breast cancer and CAD, demonstrating that individuals with higher PRS values were more likely develop these conditions [18, 19]. Moreover, individuals with higher genetic risks benefited more significantly from adhering to recommended lifestyle changes, suggesting that targeted lifestyle interventions could be particularly effective for those genetically predisposed to certain cancers, and thereby described the value of integrating PRSs with environmental factors for personalized cancer prevention strategies [20]. It is clear that, genomic prediction, which utilizes information from an individual’s genetic profile, plays a crucial role in personalized medicine [21, 22].

However, the effectiveness of PRS can be limited if environmental factors, which also play a crucial role in disease development, were not considered. Therefore, Genome Wide Environment Interaction Studies (GWEIS) offer a valuable approach to uncover how genetic predispositions interact with environmental factors, potentially improving the predictive power of PRS models by accounting for environmental contributions to disease risk [23]. Consequently, incorporating GxE into PRS models represents an important advancement in this field [24] as they account for the varying effects of environmental exposures on an individual’s health outcomes based on their genetic makeup. For instance, the effect of diet on DIAB risk could be significantly higher in individuals with certain genetic predispositions than in those without such genetic traits [25, 26]. IPRS [27] is a method that incorporates GxE in their PRS model. However, this method suffers from inflated type 1 error rate with its modeling approach [28]. Improved integration of GxE interactions in PRS models could offer more accurate and personalized risk assessments, correctly accounting for how individual genetic profiles modify responses to environmental exposures.

In this study, we aimed to investigate the GxE interactions within PRS models for complex traits and diseases. Specifically, we evaluated how specific environmental factors such as healthy diet (HD), PA, and other lifestyle variables interact with genetic variants to influence the risk of developing obesity-related traits and diseases. We employed our recently developed GxEprs method [28], designed to enhance the detection and interpretation of GxE interactions within PRS framework. This method is optimized to account for potential misspecification and reduce the likelihood of false-positive findings. By leveraging this advanced statistical method, we aimed to uncover new insights into the dynamic interplay between genes and the environmental factors.

## Methods

### Genotype data and quality control process in the UK biobank

We utilized genotype data from the UK Biobank, a cohort comprising over 500,000 individuals from the UK [29], genotyped using the UK Biobank Axiom Array and imputed with the Haplotype Reference Consortium and UK10K + 1000 Genomes reference panels [30]. We applied the same quality control procedures as detailed in our previous study[28], focusing on the White British population to minimize genetic heterogeneity. After quality control, 1,118,829 SNPs and 288,792 individuals were retained for analysis, using HapMap3 SNPs for robust genetic prediction [28].

### Sample sizes

We randomly split the total number of individuals into 2 datasets in the ratio of 8:2, namely discovery and target datasets. The discovery dataset (231,034 individuals) was used to perform GWEIS and obtain the estimated SNP effects. The target dataset (57,758 individuals) was used for construction of PRSs using the estimated SNP effects projected by the discovery dataset, and for real data analysis utilizing genomic prediction models as explained in subsequent sections.

### Phenotypes

The selected phenotypes include four commonly known obesity-related complex traits such as body mass index (BMI), waist-to-hip ratio (WHR), body fat percentage (BF) and waist circumference (WC), all of which were analysed as quantitative traits. In addition, we considered nine other conditions as binary traits. These included cardio-vascular diseases such as hypertension (HYP), stroke (STRO) and coronary artery disease (CAD), metabolic diseases such as diabetes (DIAB) and thyroid disorders (THY), mental disorders such as depression (DEPR), cancers such as obesity-related-hormone-sensitive cancer (OHCAN), eye disorders such as cataract (CATA), and gastrointestinal disorders such as hernia (HERN). Notably, we considered incident cases in this study, to make sure that the environmental exposure has occurred before the diagnosis. Consequently, the total sample size was reduced by omitting participants diagnosed with a particular disease before the baseline period as defined in the UK Biobank (see Table S4 for details).

These traits were considered due to their significant relevance to, or strong association with, obesity [31–44], aiming to provide novel insights to obesity-related health challenges.

### Environmental variables and fixed effects

To account for non-genetic environmental effects, we included fixed effects such as sex, age at recruitment, Townsend deprivation index (TDI), and education in years[45] in our analysis. Additionally, to control for potential confounding due to population stratification, we also adjusted for the first 10 genetic principal components derived from the genome-wide data [46]. In the discovery dataset, the phenotype (outcome) was adjusted during the GWEIS stage. In the target dataset, these confounders were included in the respective target models to capture their effects on the outcome.

In the GxE analysis, we considered several environmental variables. For quantitative outcomes (BMI, WHR, BF or WC), we examined five lifestyle factors: healthy diet (HD), neuroticism score (NS), physical activity (PA), pure alcohol consumption (PALC) [47] and pack-years of smoking (SMK) as the **E** variable in GxE. For binary outcomes (HYP, STRO, CAD, DIAB, THY, DEPR, OHCAN, CATA or HERN), we examined nine environmental variables including the four quantitative outcomes (BMI, WHR, BF and WC) and the five lifestyle factors (HD, NS, PA, PALC and SMK) as the **E** variable in GxE. Each outcome was analyzed with its corresponding environmental variables. It was important to note that these environmental variables were standardized independently in the discovery and target datasets before being used in the downstream analysis.

For a comprehensive overview of the variables used in our study, including detailed information on the adjusted phenotypes and environmental variables, see Tables S1– S5.

### Model Equations

Our study utilizes advanced modeling approaches as developed by Jayasinghe et al. [28]. We denote those models proposed for quantitative traits and binary traits as GxEprs_QT (equivalent to Model 4 in ref. [28]) and GxEprs_BT (equivalent to Model 5 in ref. [28]) respectively, as shown below.

For quantitative traits, the GxEprs_QT model (linear) is specified as:

GxEprs_QT $${{{\bf{y}}}}=\hat{{\alpha }_{1}}{\hat{{{{\bf{X}}}}}}_{{{{\bf{add}}}}}+\hat{{\alpha }_{2}}{{{\bf{E}}}}+\hat{{\alpha }_{3}}{\hat{{{{\bf{X}}}}}}_{{{{\bf{gxe}}}}}\odot {{{\bf{E}}}}+\hat{{\alpha }_{4}}{\hat{{{{\bf{X}}}}}}_{{{{\bf{gxe}}}}}+{{{\boldsymbol{\varepsilon }}}}$$

where **y** represents the phenotype, ${\hat{{{{\bf{X}}}}}}_{{{{\bf{add}}}}}$ and ${\hat{{{{\bf{X}}}}}}_{{{{\bf{gxe}}}}}$ are the PRSs based on the main additive and interaction effects estimated in the discovery GWEIS, **E** is the environmental variable, and ${\hat{{{{\bf{X}}}}}}_{{{{\bf{gxe}}}}}\odot {{{\bf{E}}}}$ represents the GxE interaction term, which combines ${\hat{{{{\bf{X}}}}}}_{{{{\bf{gxe}}}}}$ with the environmental variable **E,** *α*_1_, *α*_2_, *α*_3_ and *α*_4_ are the estimated regression coefficients for ${\hat{{{{\bf{X}}}}}}_{{{{\bf{add}}}}}$, **E,** ${\hat{{{{\bf{X}}}}}}_{{{{\bf{gxe}}}}}\odot {{{\bf{E}}}}$ (interaction) and ${\hat{{{{\bf{X}}}}}}_{{{{\bf{gxe}}}}}$ respectively, and ***ε*** is the residual. GxEprs_QT* (equivalent to Model 4* in ref. [28]) is a variant of GxEprs_QT, in which only the GxE interaction component (${\hat{{{{\bf{X}}}}}}_{{{{\bf{gxe}}}}}\odot {{{\bf{E}}}}$) is permuted, while the correlation structure between the outcome variable and other model components remains unchanged. Additionally, we refer to the GxEprs_QT model without the interaction component as GxEprs_QT_reduced.

For binary traits, the GxEprs_BT model (logistic) is specified as:

GxEprs_BT $${{{\rm{logit}}}}\left(P({{{\bf{y}}}}=1| {\hat{{{{\bf{X}}}}}}_{{{{\rm{add}}}}},{\hat{{{{\bf{X}}}}}}_{{{{\rm{gxe}}}}},{{{\bf{E}}}})\right)=\hat{{\alpha }_{1}}{\hat{{{{\bf{X}}}}}}_{{{{\bf{add}}}}}+\hat{{\alpha }_{2}}{{{\bf{E}}}}+\hat{{\alpha }_{3}}{\hat{{{{\bf{X}}}}}}_{{{{\bf{gxe}}}}}\odot {{{\bf{E}}}}+\hat{{\alpha }_{4}}{\hat{{{{\bf{X}}}}}}_{{{{\bf{gxe}}}}}+\hat{{\alpha }_{5}}{{{{\bf{E}}}}}^{2}$$

where variables are defined as in GxEprs_QT, and $\hat{{\alpha }_{5}}$ is the estimated regression coefficient for quadratic effects of **E**. Note that the GxEprs_BT model is specified within a logistic regression framework to model binary outcomes. In this formulation, a quadratic term for the environmental variable was included to account for potential model misspecification. In contrast, the GxEprs_QT model, used for continuous traits, did not include a quadratic term, as the linear formulation was sufficient and preferred for maintaining model parsimony [28]. GxEprs_BT* (equivalent to Model 5* in ref. [28]) is a variant of GxEprs_BT, in which only the GxE interaction component (${\hat{{{{\bf{X}}}}}}_{{{{\bf{gxe}}}}}\odot {{{\bf{E}}}}$) is permuted, while the correlation structure between the outcome variable and other model components remains unchanged. Additionally, we refer to the GxEprs_BT model without the interaction component as GxEprs_BT_reduced.

### Phase I: separate environmental variable analysis

In the first phase, we analyzed each quantitative and binary trait separately, with their respective environmental variables, using the aforementioned GxE PRS models and these analyses were performed using the R package “GxEprs”. From this, we aimed to identify the significant GxE interactions to capture significant GxE signals. Then we evaluated the model performance by computing the difference between coefficient of determination (*R*^2^) of the nested models GxEprs_QT and GxEprs_QT_reduced for quantitative traits, and the difference between area under the receiver operating characteristic (ROC) curve (AUC) values of the nested models GxEprs_BT and GxEprs_BT_reduced for binary traits, using the R packages “r2redux” [48] and “R2ROC” [49] respectively.

### Phase II: composite environmental variable analysis

In the second phase, we aimed to enhance statistical power of the study by reducing dimensionality and summarizing multiple correlated environmental variables into a single composite measure, using two approaches. In the first technique, we combined the standardized environmental variables by simple summation assuming uniform weights, but distinct directions that aids to follow healthy lifestyle and constructed a single vector (denoted by ‘sum_dir’ throughout the text). Here we considered HD and PA as positively contributed and the rest of the environmental variables as negatively contributed to a healthy lifestyle, in order to make the aggregation more meaningful. In the second technique, we combined the environmental variables as a weighted summation. We used the first principal component of the environmental variables as the composite E variable, where the weights of each standardised environmental variable were determined by the loadings of the first principal component, obtained using the “princomp” function in R. This constructed a single vector to fit with each phenotype (denoted by ‘PC1’ throughout the text). These two techniques were employed for all the respective environmental variables, analyzed with quantitative and binary phenotypes separately. Similar to the first phase, we aimed to capture significant GxE signals, when a composite environmental variable was considered, and finally evaluated the model performances using the respective R packages as described previously.

### Phase III: selected environmental variable analysis

In this phase, we extended the experimental design incorporated in the first two phases into a multiple environmental variable context. We utilized the PRSs constructed in the first phase and employed them in the same target model, given the phenotype. We followed a Step-wise selection procedure (forward selection algorithm) to select the most important environmental variable -related variables using the R package MASS, which uses Akaike Information Criterion (AIC) for the process. Then we recorded the significance of selected variables through regression summary outputs. For quantitative traits (BF, BMI, WC and WHR) we applied a forward selection technique to select across **X**_**gxe**_ ⊙ **E,** **X**_**add**_, **X**_**gxe**_, and **E** where HD, NS, PA, PALC and SMK were considered as **E**. For binary traits (CAD, CATA, DEPR, DIAB, HERN, HYP, OHCAN, STRO and THY), we also considered **E**^2^ in the pool of featured variables. This allowed us to account for correlation structures between all the environmental variable -specific model components simultaneously, which was more informative than incorporating environmental variables one at a time (phase I) or composite environmental variable method (phase II).

In this study, we utilize the GxEprs models to explore significant GxE and assess their contribution to the variance explained in phenotypic traits related to obesity. The study is structured into three phases, each designed to examine different configurations of environmental variables within the genomic prediction context. Phase I focuses on the effects of individual environmental variables at a time, Phase II integrates these variables into a composite environmental variable, and Phase III assesses the overall predictive power of a model that includes a set of optimally selected environmental variables. This phased approach demonstrates how the GxEprs models can be applied to enhance understanding and improve the accuracy of existing genomic predictions by incorporating GxE component. Our objective is to provide a methodological framework for researchers to apply the GxEprs models according to their specific research questions and contexts, without recommending one design phase over another as superior.

## Results

### Phase I: separate environmental variable analysis

**Fig. 1:**
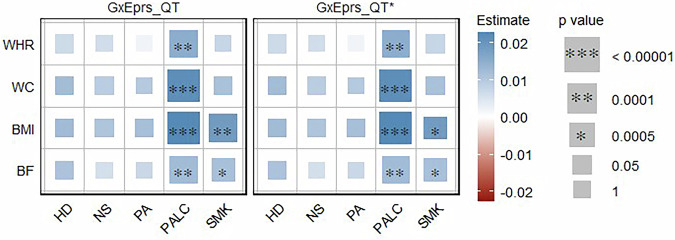
Estimates and significance of GxE components for quantitative phenotypes across environmental variables when fitting GxE PRS models. The heatmap represents the estimated regression coefficient of the GxE term for each model. From the total of 288,792 individuals, 80% (~231,034 individuals) formed the discovery sample, while the remaining 20% (~57,758 individuals) comprised the target sample. Dark red to dark blue reflects the transition from negative to positive associations, indicating the magnitude of the regression coefficient. The size of each square in the heatmap was proportional to the corresponding *p* value. Significance levels were indicated by asterisks, representing the significance after Bonferroni correction (significance level = 0.05/101~0.0005), considering a total of 101 analyses conducted. The number of permutations performed in GxEprs_QT* was determined based on the *p* value obtained in GxEprs_QT to ensure an adequate number of permutations (with a minimum of 1000). The horizontal axis represents the environmental variables involved in the GxE component, while the vertical axis represents the phenotypes. We have included all the confounders as fixed components in the discovery and target models

We analyzed all quantitative traits (BF, BMI, WC and WHR) with corresponding environmental variables (HD, NS, PA, PALC and SMK) applying models GxEprs_QT and GxEprs_QT* as previously proposed [28]. Results indicated that for all of the quantitative traits, genetic effects were modulated by PALC. Similarly for BMI and BF, genetic effects were modulated by SMK (Fig. 1). The estimated regression coefficients, along with their corresponding standard errors, test statistics, and p values for the quantitative traits with the relevant modifiable environmental variables, were available in Table S6.

Moreover, *R*^2^ values obtained by fitting GxEprs_QT and GxEprs_QT_reduced models across each quantitative phenotype/ environmental variable pair analyzed, along with the respective p values for their difference (*ΔR*^2^) are shown in Table S7. The proportion of variance explained by GxE component (Var(GxE)) ranged between 0.1–0.8 % across each quantitative phenotype/ environmental variable pair. For the majority of quantitative traits, the GxE component explained a modest but significantly significant proportion of model variance, and hence, enhanced the models’ overall predictive ability. Regardless of the environmental variable regressed with the phenotype, both GxEprs_QT and GxEprs_QT_reduced reported relatively higher *R*^2^ values that were  ~50% when fitted with BF and WHR (Table S7).

**Fig. 2:**
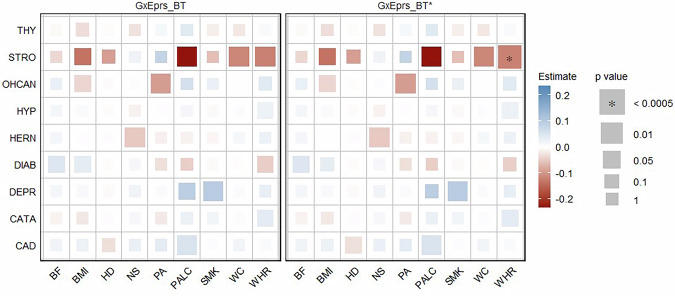
Estimates and significance of GxE components for binary phenotypes across environmental variables when fitting GxE PRS models. The heatmap represents the estimated regression coefficient of the GxE term for each model. From the total of 288,792 individuals, 80% (~231,034 individuals) formed the discovery sample, while the remaining 20% (~57,758 individuals) comprised the target sample. Dark red to dark blue reflects the transition from negative to positive associations, indicating the magnitude of the regression coefficient. The size of each square in the heatmap was proportional to the corresponding p value. Significance levels were indicated by asterisks, representing the significance after Bonferroni correction (significance level = 0.05/101~0.0005), considering a total of 101 analyses conducted. The number of permutations performed in GxEprs_BT* was determined based on the p value obtained in GxEprs_BT to ensure an adequate number of permutations (with a minimum of 1000). The horizontal axis represents the environmental variables involved in the GxE component, while the vertical axis represents the phenotypes. We have included all the confounders as fixed components in the discovery and target models

We also analyzed all binary traits (CAD, CATA, DEPR, DIAB, HERN, HYP, OHCAN, STRO and THY) with corresponding environmental variables (HD, NS, PA, PALC, SMK, BF, BMI, WC and WHR) applying models GxEprs_BT and GxEprs_BT* as previously proposed [28]. Results shown in Fig. 2 indicated no significant GxE signals when GxEprs_BT was fitted for binary traits. Only the GxE component for STRO/WHR was marginally insignificant (p value = 6.62E–04) which explained the significance captured by GxEprs_BT*. The estimated regression coefficients, along with their corresponding standard errors, test statistics, and p values for the binary traits with the relevant modifiable environmental variables, are provided in Table S8.

AUC values for models fitted for binary traits, with and without the GxE component (GxEprs_BT and GxEprs_BT_reduced models), p values for difference in AUCs (*Δ*AUC), and the computed variance explained by GxE components are shown in Table S9. Additionally, we computed Var(GxE) across each binary phenotype/ environmental variable pair. GxEprs_BT did not explain a statistically significant proportion of variance than that of GxEprs_BT_reduced in many instances (p value  < 0.05). Despite the statistical significance of AUC increment, when we quantified the Var(GxE), we found a small proportion of additional variability explained by the GxE component ranging from 0 to 5.1%, across each binary phenotype/ environmental variable pair. However, the overall AUC of HYP, CAD, DIAB and CATA, across all environmental variables were greater than 70%, indicating fair to good discriminatory power in these clinical applications, as supported by literature [50, 51], regardless of whether GxE component was in the model or not. Notably, the lowest AUCs were observed for OHCAN.

### Phase II: composite environmental variable analysis

**Fig. 3:**
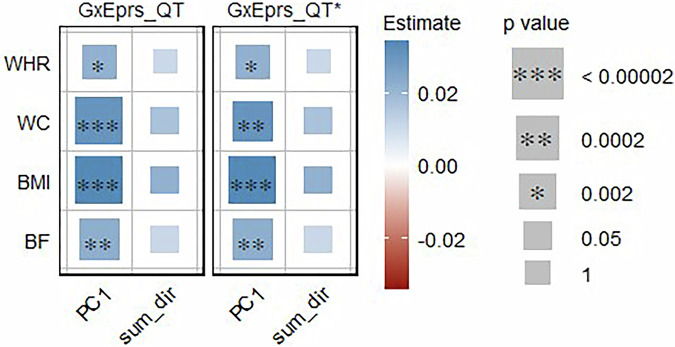
Estimates and significance of GxE components for quantitative phenotypes/composite environmental variables when fitting GxE PRS models. The heatmap represents the estimated regression coefficient of the GxE term for each model. From the total of 288,792 individuals, 80% (~231,034 individuals) formed the discovery sample, while the remaining 20% (~57,758 individuals) comprised the target sample. Dark red to dark blue reflects the transition from negative to positive associations, indicating the magnitude of the regression coefficient. The size of each square in the heatmap was proportional to the corresponding p value. Significance levels were indicated by asterisks, representing the significance after Bonferroni correction (significance level = 0.05/26~0.002), considering a total of 26 analyses conducted. The number of permutations performed in GxEprs_QT* was determined based on the p value obtained in GxEprs_QT to ensure an adequate number of permutations (with a minimum of 1000). The horizontal axis represents the composite environmental variables involved in the GxE component, while the vertical axis represents the phenotypes. We have included all the confounders as fixed components in the discovery and target models. PC1: the composite variable based on weighted summation of each environmental variable. Sum_dir: the composite variable based on unweighted summation of each environmental variable, accounted for direction in relation to healthy lifestyle

We analyzed all quantitative traits (BF, BMI, WC and WHR) with corresponding composite environmental variables (sum_dir: the composite variable based on unweighted summation of each environmental variable, accounted for direction in relation to healthy lifestyle and PC1: the composite variable based on weighted summation of each environmental variable as defined in Phase II of Methods section) constructed by combining the environmental variables HD, NS, PA, PALC and SMK, applying models GxEprs_QT and GxEprs_QT*. Figure 3 provides evidence that genetic effects of all quantitative phenotypes were modulated by weighted composite environmental variable (PC1), while sum_dir did not indicate any significant signal. Therefore, when the considered environmental variables were combined using appropriate weights, the composite variable behaves as a modulating variable of genetic effects for all the quantitative traits considered. The estimated regression coefficients, along with their corresponding standard errors, test statistics, and p values for the quantitative traits with the relevant modifiable composite environmental variables, are available in Table S10.

When considering the composite environmental variable PC1, a statistically significant improvement in *R*^2^ was noted for all quantitative traits when comparing models with and without the GxE interaction term (p value  < 0.05), while for many instances when composite environmental variable was sum_dir, showed the same, as demonstrated in Table S11. Overall, the Var(GxE) ranged from 0.1% to 0.9% across quantitative phenotype/composite environmental variable pairs. These results closely aligned with those discussed in Phase I (Table S7), across each quantitative trait. Hence, regardless of the type of composition of the environmental variable, both GxEprs_QT and GxEprs_QT_reduced reported relatively higher *R*^2^ values that were  ~ 50% when fitted with BF and WHR. Consequently, the prediction accuracy for quantitative traits appears consistent, showing no considerable differences attributable to the design choices made in either phases I or II.

**Fig. 4:**
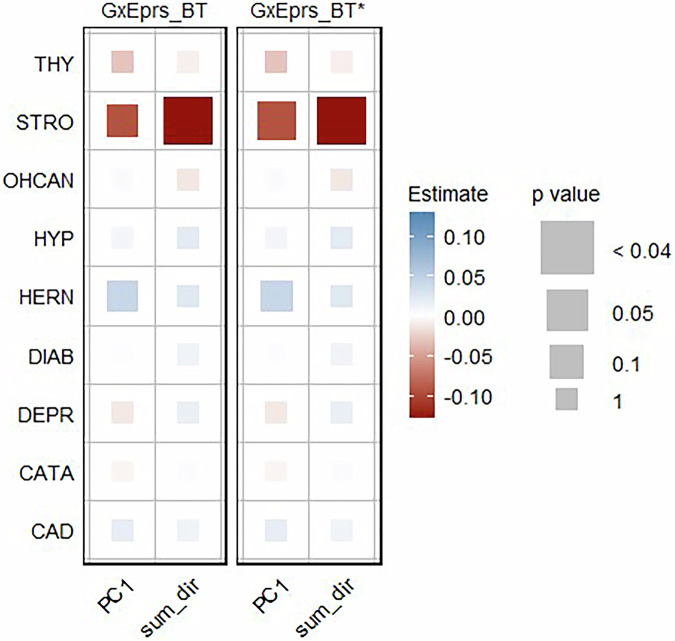
Estimates and significance of GxE components for binary phenotypes/composite environmental variables when fitting GxE PRS models. The heatmap represents the estimated regression coefficient of the GxE term for each model. From the total of 288,792 individuals, 80% (~231,034 individuals) formed the discovery sample, while the remaining 20% (~57,758 individuals) comprised the target sample. Dark red to dark blue reflects the transition from negative to positive associations, indicating the magnitude of the regression coefficient. The size of each square in the heatmap was proportional to the corresponding p value. Significance levels were indicated by asterisks, representing the significance after Bonferroni correction (significance level = 0.05/26~0.002), considering a total of 26 analyses conducted. The number of permutations performed in GxEprs_BT* was determined based on the p value obtained in GxEprs_BT to ensure an adequate number of permutations (with a minimum of 1000). The horizontal axis represents the composite environmental variables involved in the GxE component, while the vertical axis represents the phenotypes. We have included all the confounders as fixed components in the discovery and target models. PC1: the composite variable based on weighted summation of each environmental variable. Sum_dir: the composite variable based on unweighted summation of each environmental variable, accounted for direction in relation to healthy lifestyle

All the binary traits (CAD, CATA, DEPR, DIAB, HERN, HYP, OHCAN, STRO and THY) were analyzed with corresponding environmental variables (sum_dir and PC1) constructed by combining the environmental variables HD, NS, PA, PALC, SMK, BF, BMI, WC, and WHR applying models GxEprs_BT and GxEprs_BT*. As shown in Fig. 4 no statistically significant GxE effects were found across any of the binary traits when considering either composite environmental variable, PC1 or sum_dir. The estimated regression coefficients, along with their corresponding standard errors, test statistics, and p values for the binary traits with the relevant modifiable composite environmental variables, were available in Table S12.

In general, there was no statistically significant improvement in AUC, (p value  < 0.05) when considering the GxE component through GxEprs_BT model, compared to GxEprs_BT_reduced model (Table S13). However, we found a small proportion of additional variability explained by the GxE component (Var(GxE)) ranging from 0 to 3.7%, across the binary phenotype/composite environmental variable pairs. Interestingly, regardless of the GxE PRS model fitted, AUC greater than 70% for traits such as CAD, CATA, DIAB, HYP, STRO and THY implying that the models may be applicable in the clinical setting, as supported by literature [50, 51].

### Phase III: selected environmental variable analysis

In this phase, we extended the models used in Phase I by incorporating multiple environmental variables selected through model selection to account for potential correlation structures between environmental variables (see “Methods”).

**Fig. 5:**
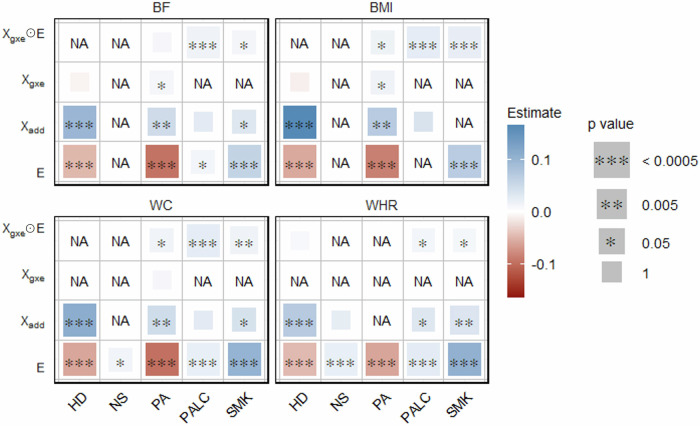
Estimates and significance of all model components for quantitative phenotypes/all environmental variables when fitting GxE PRS models. The heatmap represents the estimated regression coefficient of all the 20 model components of which the step-wise selection was applied to. From the total of 288,792 individuals, 80% (~231,034 individuals) formed the discovery sample, while the remaining 20% (~57,758 individuals) comprised the target sample. Dark red to dark blue reflects the transition from negative to positive associations, indicating the magnitude of the regression coefficient. The size of each square in the heatmap was proportional to the corresponding p value. Significance levels were indicated by asterisks. The horizontal axis represents the environmental variables, while the vertical axis represents the model components corresponding to each of the environmental variable considered. The variables that were removed in the step-wise procedure were indicated as NA. Each block represents quantitative phenotypes as the response variable. We have included all the confounders as fixed components in the discovery and target models

The results shown in Fig. 5 mostly align with those from phase I (see Fig. 1), with regards to our key parameter the GxE component. Additionally, the results show that genetic effects of BMI and WC were modulated by PA, a relationship not observed in Phase I. This maybe due to the correlation structures accounted for in this phase, which were previously ignored.

Using GxEprs models facilitating multiple environmental variables simultaneously through model selection, we explored the overall prediction accuracy (*R*^2^) of each outcome, for quantitative traits (Table S14). Interestingly, *R*^2^ values showed no improvement or decline compared to previous phases. WHR and BF demonstrated relatively higher *R*^2^, while BMI had the lowest.

**Fig. 6:**
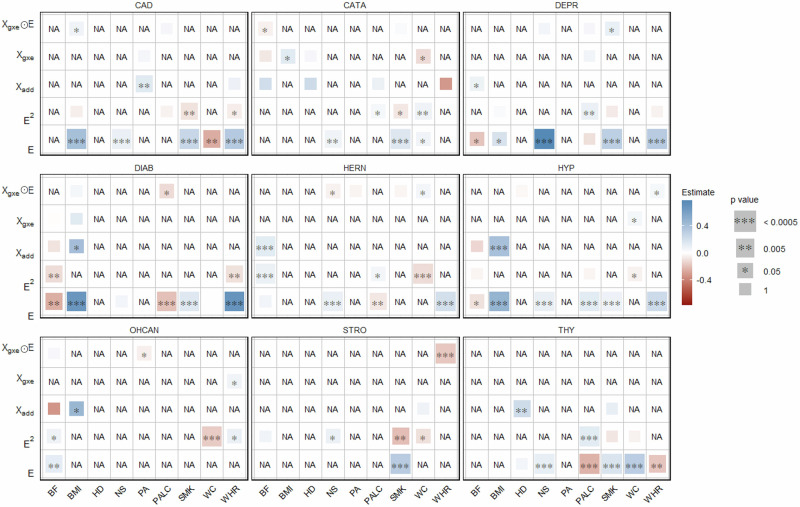
Estimates and significance of all model components for binary phenotypes/all environmental variables when fitting GxE PRS models. The heatmap represents the estimated regression coefficient of all the 45 model components of which the step-wise selection was applied to. From the total of 288,792 individuals, 80% (~231,034 individuals) formed the discovery sample, while the remaining 20% (~57,758 individuals) comprised the target sample. Dark red to dark blue reflects the transition from negative to positive associations, indicating the magnitude of the regression coefficient. The size of each square in the heatmap was proportional to the corresponding p value. Significance levels were indicated by asterisks. The horizontal axis represents the environmental variables, while the vertical axis represents the model components corresponding to each of the environmental variable considered. The variables that were removed in the step-wise procedure were indicated as NA. Each block represents binary phenotypes as the response variable. We have included all the confounders as fixed components in the discovery and target models

Figure 6 highlights several noteworthy findings for binary traits compared to previous phases. For instance, the genetic effects of CAD and CATA were influenced by BMI, and DEPR by SMK. Additionally, the genetic effects of DIAB were modified by PALC, HERN by NS and WC, and OHCAN by PA. WHR had a significant effect on modifying the genetic effects of HYP and showed a strong, highly significant influence on STRO.

Consistent with our approach for quantitative traits in Phase III, we evaluated the overall model prediction accuracy for binary traits, using AUC values for each final selected model (see Table S15). HYP, CAD, DIAB, DEPR, and CATA achieved AUC values above 70%. In general, this observation was well-aligned with AUCs reported in Phase I, except for DEPR. As observed in Phase I, OHCAN reported the lowest AUC value.

Similar to the analysis of quantitative traits under Phase III, we computed the overall model prediction accuracy for binary traits in terms of AUC values for each of the finally selected model (Table S15). HYP, CAD, DIAB, DEPR and CATA reported AUC values greater than 70%. Similar to Phase I, the lowest AUC value was observed for OHCAN.

Additionally, we would highlight that the inclusion of confounding variables to the prediction model played an important role. For instance, the highest variability across all components fitted for quantitative traits was explained by the confounder ‘sex’ (Notes S1–S4). Furthermore, we found that the confounder ‘age’ was the most important variable when predicting binary traits, except for DEPR (Notes S5–S13). However, when predicting the risk of developing DEPR, NS was found to be the most important, reporting the highest significance (Note S7).

## Discussion

In this study, we aimed to explore the significance of GxE interactions in the context of obesity-related complex traits and diseases. By employing the GxEprs method in a multi-phase analysis strategy, we investigated the effectiveness of various modeling approaches to capture significant GxE signals.

In Phase I, we applied the GxEprs model [28] and observed significant GxE signals for quantitative traits such as BMI, WHR, WC and BF. This finding aligns with previous studies [9–11, 52] which reported significant GxE interactions for those traits, supporting the robustness of our results. However, we found no significant GxE signals for binary traits. The absence of significant GxE effects for binary traits such as HYP contrasts with findings from the GxEsum model [12], which was based on prevalent cases, whereas our study focused on incident cases. This difference in study design may explain the contrasting results, as incident cases ensure that environmental factors precede the onset of conditions like HYP. However, the smaller sample size of incident cases compared to prevalent cases may limit statistical power, possibly masking certain GxE effects. To enhance power, we introduced a composite variable approach in the subsequent phase, but this approach still did not yield significant GxE signals for binary traits. In Phase III, we expanded our analysis by incorporating multiple environmental variables in a single model to account for correlations between them, providing a more comprehensive approach than Phase I. Overall, the results from Phase III were consistent with those from Phase I, demonstrating the robustness and reliability of our findings across modeling phases.

Our study identified significant GxE signals at each phase, capturing modifiable environmental variables for various obesity-related complex traits and diseases using the proposed GxEprs models. Notably, in Phase I, we found that the genetic effects of BF, BMI, WC, and WHR were modified by PALC, while BF and BMI were also modified by SMK. In Phase III, when we included multiple environment-related variables selected through a step-wise approach in the same model, PALC and SMK were found to modify BF, BMI, WC, and WHR, while PA modified BMI and WC. Interestingly, the direction of the **E** variable coefficient estimates for quantitative traits aligned with expectations—for instance, HD and PA were negatively associated with obesity-related traits, while NS, PALC, and SMK showed positive associations, implying the importance of a healthy lifestyle in reducing obesity. In contrast, for binary traits, significant GxE signals were only found in Phase III, with a few unexpected associations such as PALC being negatively associated with DIAB, HERN, and THY. These unexpected findings may be due to potential collider biases, given the complex correlation structure of environmental factors and/or lack of prior knowledge on causal direction in modeling.

It is well-known that GWEIS test statistics can frequently exhibit inflation [53]. Therefore, we assessed GWEIS model specifications using diagnostic tools such as genomic QQ plots, Manhattan plots and inflation factors including the genomic inflation factor (*λ*), the scaled genomic inflation factor (*λ*_1000_), and the theoretical inflation factor (lapprox), for the outcome/environmental variable pairs that reported significant GxE interaction in any phase (Phase I - III). The genomic inflation factor reflects the median observed chi-square test statistic relative to the expected median under the null, while the scaled version adjusts this value to a standard sample size of 1000 to facilitate comparisons across studies with differing sample sizes [54]. The theoretical inflation factor (available from the “lapprox” function in the R package “stmgp”), proposed by Ueki et al. [53], assumes no population stratification and unrelated individuals, and serves as a useful diagnostic to quantify model misspecification. The majority of GWEIS models appeared appropriately specified; however, the DIAB/PALC pair showed slight to moderate inflation under the null (lapprox = 1.226, see Table S16 for full results). Since model misspecification can impact the accuracy of SNP effect estimates and, consequently, PRS reliability, we emphasize the importance of careful diagnostic evaluation prior to downstream analyses.

The identification of significant GxE interactions in this study may offer insight that could eventually inform public health strategies. By exploring how specific environmental exposures may interact with genetic factors to influence disease risk, our general approach suggests potential pathways for targeted interventions. GxEprs models may assist in more accurate risk computations, than previously developed methods that preceded GxEprs. However, it is important to note that the clinical application of genomic prediction models, including those for diseases like obesity, remains primarily within the research domain. While GxEprs models could one day assist in precise risk computations and possibly aid in early diagnosis by considering both genetic and environmental factors, these applications are not yet directly transferable to clinical settings. Personalized health advice and treatment optimization based on GxE interactions hold promise but require further validation. For example, our results may inform how environmental conditions could mitigate the risk for individuals with a high PRS for obesity, yet practical, evidence-based approaches in clinical environments are needed to realize these benefits fully. This study underscores the potential of GxE research to contribute to personalized medicine, although significant work remains to translate these findings into clinical practice.

In addition to identifying significant GxE signals, we quantified the proportion of variance explained by corresponding GxE components at the population level, and we noticed that the percentage contribution of GxE were generally quite modest. This observation aligns with findings from Zhou et al. [11], who demonstrated using a whole-genome approach that lifestyle factors can significantly modulate the genetic and nongenetic variance components of cardiovascular traits. As illustrated in their study, variance estimates of interactions vary among different stratified groups based on environmental exposures (Fig. 1 (ref. [11])). This suggests that the environmental conditions included in a study could substantially influence the variance attributable to GxE interactions. If the environmental conditions are more varied and extreme, they might expose stronger interaction effects that are not detectable under more moderate or uniform conditions. Precisely, in extreme environmental conditions, this Var(GxE) could reach considerably higher values, which could be clearly a matter of fact in the clinical setting. However, the utilization of our findings into clinical practice must be approached with caution due to several limitations. The modest improvements in predictive accuracy, while statistically significant, may require clinical validation to confirm their relevance in practical settings. Consequently, the models that we adopted do not guarantee causal directions. Finally, the study’s focus on a primarily White British population may limit the generalizability of the findings to other ethnic groups. Lifestyle interventions can be more effectively tailored by integrating GxE information, allowing healthcare providers to target individuals with modifiable environmental risks, such as poor diet or physical inactivity, in conjunction with their genetic susceptibility. At a broader level, GxE can inform public health policies by identifying population subgroups at higher risk due to specific genetic-environmental interactions, leading to more efficient resource allocation in preventive efforts. Ultimately, GxE interaction enhances the clinical utility of PRS in managing obesity, allowing for more personalized, effective prevention, and treatment strategies.

We remark some methodological limitations identified in Phase III and potential remedies that future researchers can employ. We used the same target samples for both model fitting using stepwise-selection method and model evaluation via the metrics *R*^2^ and AUC, which might have led to an overly optimistic assessment of model performance. To mitigate this issue and align with best practices in future studies, we note the importance of using an independent test sample for final model evaluation, as recommended by Khera et al. [55]. This approach helps ensure that the results are not just a reflection of the model’s fit to a particular dataset but are indicative of its generalizability across different samples. Moreover highlight the necessity of following a suitable post-selection inference technique such as sample splitting, simultaneous inference or conditional selective inference [56], to address the issue of distorted p values, which arises when model selection process and significant variable detection are both conducted on the same target dataset.

In conclusion, our multi-phase analysis highlights the complex interplay between genetic and environmental factors in influencing complex traits and diseases. While significant GxE signals were identified for quantitative traits, the lack of significant findings for binary traits suggests potential limitations in the current methodology in relation to smaller sample sizes. The introduction of composite variables in Phase II aimed to enhance power, but challenges remained in capturing GxE signals for binary traits, if they do exist. The PC1 composite variable proved to be the most informative, emphasizing the importance of weighted schemes in future analyses. Phase III demonstrated the value of incorporating multiple environmental variables for a more comprehensive modeling. Future research should address these limitations by increasing sample sizes, particularly for binary traits, and exploring more advanced computational methods and resources. Further investigation into the discrepancies observed between different phases is essential to determine whether they were due to genuine effects or methodological constraints. Overall, our study highlights key gene-environment interactions, signifying that some genetic effects are modifiable, offering insights into the dynamics of complex traits and diseases. These findings pave the way for future studies to refine and expand upon our approaches, ultimately enhancing the understanding of GxE interactions in complex trait analysis.

## Supplementary information

Supplementary Information
