# Supplementary material for: Analyzing complex traits and diseases using GxE PRS: genotype-environment interaction in polygenic risk score models
Source: J Hum Genet. 2025 Aug 14;70(11):547–56. doi: 10.1038/s10038-025-01378-2 (PMC12552121; doi:10.1038/s10038-025-01378-2)
Supplement: Supplementary file 1 — Supplementary Information [file 10038_2025_1378_MOESM1_ESM.pdf]

Appendix

Data

Table S1: Quantitative variable details

UK biobank Field ID	Variable	Abbreviation
21001	Body mass index	BMI
20127	Neuroticism score	NS
22040	Summed MET minutes per week for all activity	PA
23099	Body fat percentage	BF
48	Waist circumference	WC
20161	Pack years of smoking	SMK
1289	Cooked vegetable intake	HD
1299	Salad/raw vegetable intake	
1309	Fresh fruit intake	
1329	Oily fish intake	
1339	Non-oily fish intake	
1379	Lamb/mutton intake	
1349	Processed meat intake	
1369	Beef intake	
1389	Pork intake	
48	Waist circumference	WHR
49	Hip circumference	
1568	Red wine intake per week	PALC
4407	Red wine intake per month	
1578	White wine/champagne intake per week	
4418	White wine/champagne intake per month	
1588	Beer/cider intake per week	
4429	Beer/cider intake per month	
1598	Spirit intake per week	
4440	Spirit intake per month	
1608	Fortified wine intake per week	
4451	Fortified wine intake per month	
5364	Other alcoholic drinks per week	
4468	Other alcoholic drinks per month	

We considered 1) fruit and vegetable intake, 2) total fish intake (oily and non-oily), 3) processed meat intake and 4) red meat intake (beef, pork and lamb/mutton) as an index of healthy diet (HD) following the American Heart Association Guidelines.[1]

1. The amount of total fruit and vegetable intake per day was obtained by combining the amount of total fruit servings (piece of fruit = 1 serving) and vegetable servings (3 tablespoons = 1 serving) consumed. Scores of individuals who had 4.5 or more servings a day were converted to 1, and the remaining scores were converted to 0.

2. Total fish intake was obtained by combining the data of oily and non-oily fish intake after re-coding weekly frequency (0 = never, 0.5 = less than once week, 1 = once a week, 3 = two to four times a week, 5.5 = five to six times a week, and 7 = once or more daily). Scores of individuals who ate 2 or more times per week were converted to 1, and remaining intake frequencies were converted to 0.

3. Total processed meat intake a week was recorded as weekly intake (0 = never, 0.5 = less than once week, 1 = once a week, 3 = two to four times a week, 5.5 = five to six times a week, and 7 = once or more daily). Scores of individuals who consumed processed meat 2 or fewer times per week were converted to 1, and the remaining intake frequencies were converted to 0.

4. Total red meat intake was obtained from combining the data for beef, lamb/mutton and pork intake based on weekly intake (0 = never, 0.5 = less than once week, 1 = once a week, 3 = two to four times a week, 5.5 = five to six times a week, and 7 = once or more

daily). The records of individuals who had 5 or fewer times per week were converted to 1, and the remaining intake frequencies were converted to 0.

The overall dietary score was derived by totaling the values for separate dietary components described above and scores ranged from a minimum of 0 point to a maximum of 4 point for each participant. In summary, a score close to 4 were represented as indicators of healthier diet habits than a score close to zero.

Waist-to-hip ratio (WHR) was computed by obtaining the ratio between waist circumference and hip circumference of corresponding individuals.

Table S2: Specifications of alcoholic drinks

**Type**	**Standard ml**	**Standard drinks**	**Alcohol %**
Red wine	750	6	12
White wine/champagne	750	6	12
Beer/cider	574	1	4
Spirit	700	25	40
Fortified wine	725	12	19
Other	275	1	5.5

We derived pure alcohol intake (PALC) variable[2] by considering the amount of drinks consumed in either weekly or monthly basis from the UK biobank field IDs shown in Table S1. We first multiplied each intake from standard milliliters (ml) per unit and alcohol percentages corresponding to each type. Then we converted weekly and monthly variables into daily variables. This quantity was then divided by standard number of drinks corresponding to each drink type. The specifications are given in Table S2. Finally, to obtain the pure alcohol in grams, we multiplied the quantity by 0.79 which is the standard for density of ethanol at room temperature,[3] and computed the row totals for each individual in the dataset.

Table S3: Binary variable details

**UK biobank Field ID**	**Variable**	**Remark**
41202	Diagnoses - main ICD10	Summary Diagnoses
41270	Diagnosis - ICD10	Summary Diagnoses
40006	Type of Cancer - ICD 10	Cancer Register
41262	Date of first in-patient diagnosis - main ICD10	Summary Diagnoses
41280	Date of first in-patient diagnosis - ICD10	Summary Diagnoses
40005	Date of Cancer Diagnosis	Cancer Register
53	Date of Attending Assessment Center	Reception
**Disease Code**	**Variable**	**Abbreviation**
E10	Insulin-dependent diabetes mellitus	DIAB
E11	Non-insulin-dependent diabetes mellitus	
E13	Other specified diabetes mellitus	
E14	Unspecified diabetes mellitus	
I10	Essential (primary) hypertension	HYP
I11	Hypertensive heart disease	
I12	Hypertensive renal disease	
I13	Hypertensive heart and renal disease	
I15	Secondary hypertension	
I22	Subsequent myocardial infarction	CAD
I23	Certain current complications following acute myocardial infarction	
I24	Other acute ischaemic heart diseases	
I25	Chronic ischaemic heart disease	
I60	Subarachnoid haemorrhage	STRO
I61	Intracerebral haemorrhage	
I62	Other nontraumatic intracranial haemorrhage	
I63	Cerebral infarction	
I64	Stroke, not specified as haemorrhage or infarction	
F32	Depressive episode	DEPR
F33	Recurrent depressive disorder	
H25	Senile cataract	CATA
H26	Other cataract	
E01	Iodine defeciency related thyroid disorders and allied conditions	THY
E02	Sub-clinical iodine deficiency hypothyroidism	
E03	Other hypothyroidism	
E04	Other non-toxic goitre	
E05	Thyrotoxicosis (hyperthyroidism)	
E06	Thyroiditis	
E07	Other disorders of thyroid	
K40	Inguinal hernia	HERN
K41	Femoral hernia	
K42	Umbilical hernia	
K43	Ventral hernia	
K44	Diaphragmatic hernia	
K45	Other abdominal hernia	
K46	Unspecified abdominal hernia	
C61	Malignant neoplasm of prostate	OHCAN
C50	Malignant neoplasm of breast	
C73	Malignant neoplasm of thyroid and other endocrine glands	
C56	Malignant neoplasm of ovary	
C54	Malignant neoplasm of corpus uteri	
C55	Malignant neoplasm of uterus, part unspecified	

Table S3 contains the details of binary scale variables used to describe disease status classification considering prevalent cases. We used ICD10 codes from UK bio bank to capture diabetes (DIAB), hypertension (HYP), coronary artery disease (CAD), stroke (STRO), depression (DEPR), cataract (CATA), thyroid disorder (THY), hernia (HERN) and obesity-related-hormone-sensitive-cancer (OHCAN) cases. We considered only the incident cases by filtering individuals who diagnosed after date attended UK bio bank test center and removing individuals who diagnosed before the date from the dataset. It is worth noting that, we derived OHCAN by combining five types of cancer, namely prostate cancer, breast cancer, thyroid cancer, ovarian cancer and uterine cancer.[4]

Table S4: Participant distribution by trait for controls and incident cases

Trait	Number of Controls	Number of Incident Cases	Total Sample Size
CAD	260,894	19,184	280,078
CATA	255,232	28,747	283,979
DEPR	271,901	14,272	286,173
DIAB	266,445	16,596	283,041
HERN	239,806	31,358	271,164
HYP	204,054	62,702	266,756
OHCAN	265,307	15,154	280,461
STRO	281,002	6,248	287,250
THY	269,395	14,443	283,838

Table S5: Other variable details

UK biobank Field ID	Variable	Remark
31	Sex	Used to adjust phenotypes
22189	Townsend deprivation index at recruitment	
21022	Age at recruitment	
6138	Qualifications	
22009	Genetic principal components	

Table S5 contains the details of all the other variables used in this study. Note that, the variable 'qualification' was based on the qualification questionnaire. We followed the International Standard Classification of Education[5] for converting the baseline qualification information to the education in years for each individual. We used the first 10 genetic principal components in our study.

Phase I: Separate Environmental Variable Analysis

Table S6: Regression summary from the GxE PRS models across each outcome/environmental variable pair for quantitative traits

Phenotype	E	Model	Component	Estimate	Standard Error	Test Statistic	P value
BMI	HD	GxEprs_QT	$\hat{\alpha}_1$	-6.24E-02	4.26E-03	-1.46E+01	2.14E-48
			$\hat{\alpha}_2$	2.61E-01	4.00E-03	6.54E+01	0.00E+00
			$\hat{\alpha}_4$	-8.83E-03	4.01E-03	-2.20E+00	2.77E-02
			$\hat{\alpha}_3$	1.25E-02	4.14E-03	3.02E+00	2.56E-03
		GxEprs_QT*	$\hat{\alpha}_3$	NA	NA	NA	2.00E-03
	PA	GxEprs_QT	$\hat{\alpha}_1$	-9.51E-02	5.99E-03	-1.59E+01	1.26E-56
			$\hat{\alpha}_2$	2.56E-01	4.39E-03	5.83E+01	0.00E+00
			$\hat{\alpha}_4$	9.33E-03	4.38E-03	2.13E+00	3.33E-02
			$\hat{\alpha}_3$	1.17E-02	5.96E-03	1.96E+00	4.96E-02
		GxEprs_QT*	$\hat{\alpha}_3$	NA	NA	NA	3.00E-02
	PALC	GxEprs_QT	$\hat{\alpha}_1$	1.66E-02	4.46E-03	3.73E+00	1.89E-04
			$\hat{\alpha}_2$	2.46E-01	4.22E-03	5.84E+01	0.00E+00
			$\hat{\alpha}_4$	2.93E-03	4.22E-03	6.95E-01	4.87E-01
			$\hat{\alpha}_3$	2.27E-02	4.20E-03	5.41E+00	6.39E-08
		GxEprs_QT*	$\hat{\alpha}_3$	NA	NA	NA	0.00E+00
	SMK	GxEprs_QT	$\hat{\alpha}_1$	6.74E-02	4.95E-03	1.36E+01	3.70E-42
			$\hat{\alpha}_2$	2.51E-01	4.39E-03	5.71E+01	0.00E+00
			$\hat{\alpha}_4$	3.55E-03	4.72E-03	7.53E-01	4.52E-01
			$\hat{\alpha}_3$	1.89E-02	4.76E-03	3.98E+00	7.03E-05
		GxEprs_QT*	$\hat{\alpha}_3$	NA	NA	NA	2.50E-04
	NS	GxEprs_QT	$\hat{\alpha}_1$	9.44E-03	4.71E-03	2.00E+00	4.51E-02
			$\hat{\alpha}_2$	2.59E-01	4.45E-03	5.83E+01	0.00E+00
			$\hat{\alpha}_4$	-9.46E-03	4.43E-03	-2.14E+00	3.27E-02
			$\hat{\alpha}_3$	1.07E-02	4.62E-03	2.32E+00	2.05E-02
		GxEprs_QT*	$\hat{\alpha}_3$	NA	NA	NA	2.20E-02
WHR	HD	GxEprs_QT	$\hat{\alpha}_1$	-5.86E-02	3.26E-03	-1.80E+01	6.30E-72
			$\hat{\alpha}_2$	1.66E-01	3.01E-03	5.51E+01	0.00E+00
			$\hat{\alpha}_4$	1.49E-03	3.01E-03	4.94E-01	6.21E-01
			$\hat{\alpha}_3$	6.85E-03	3.17E-03	2.16E+00	3.09E-02
		GxEprs_QT*	$\hat{\alpha}_3$	NA	NA	NA	1.70E-02
	PA	GxEprs_QT	$\hat{\alpha}_1$	-6.84E-02	4.26E-03	-1.61E+01	7.09E-58
			$\hat{\alpha}_2$	1.59E-01	3.29E-03	4.84E+01	0.00E+00
			$\hat{\alpha}_4$	-8.04E-04	3.27E-03	-2.46E-01	8.06E-01
			$\hat{\alpha}_3$	1.91E-03	4.24E-03	4.49E-01	6.53E-01
		GxEprs_QT*	$\hat{\alpha}_3$	NA	NA	NA	9.54E-01
	PALC	GxEprs_QT	$\hat{\alpha}_1$	4.31E-02	4.05E-03	1.06E+01	2.32E-26
			$\hat{\alpha}_2$	1.57E-01	3.22E-03	4.87E+01	0.00E+00
			$\hat{\alpha}_4$	3.12E-03	3.22E-03	9.70E-01	3.32E-01
			$\hat{\alpha}_3$	1.51E-02	3.89E-03	3.89E+00	9.96E-05
		GxEprs_QT*	$\hat{\alpha}_3$	NA	NA	NA	7.00E-05
	SMK	GxEprs_QT	$\hat{\alpha}_1$	1.13E-01	4.09E-03	2.76E+01	8.76E-167
			$\hat{\alpha}_2$	1.56E-01	3.28E-03	4.77E+01	0.00E+00
			$\hat{\alpha}_4$	-4.05E-04	3.39E-03	-1.19E-01	9.05E-01
			$\hat{\alpha}_3$	7.66E-03	3.96E-03	1.93E+00	5.32E-02
		GxEprs_QT*	$\hat{\alpha}_3$	NA	NA	NA	2.30E-02
	NS	GxEprs_QT	$\hat{\alpha}_1$	3.09E-02	3.35E-03	9.21E+00	3.39E-20
			$\hat{\alpha}_2$	1.61E-01	3.29E-03	4.89E+01	0.00E+00
			$\hat{\alpha}_4$	-3.22E-03	3.29E-03	-9.78E-01	3.28E-01
			$\hat{\alpha}_3$	6.27E-03	3.28E-03	1.91E+00	5.61E-02
		GxEprs_QT*	$\hat{\alpha}_3$	NA	NA	NA	5.60E-02
BF	HD	GxEprs_QT	$\hat{\alpha}_1$	-5.32E-02	3.27E-03	-1.63E+01	2.22E-59
			$\hat{\alpha}_2$	1.99E-01	3.02E-03	6.61E+01	0.00E+00
			$\hat{\alpha}_4$	-6.91E-03	3.01E-03	-2.30E+00	2.16E-02
			$\hat{\alpha}_3$	1.10E-02	3.18E-03	3.47E+00	5.28E-04
		GxEprs_QT*	$\hat{\alpha}_3$	NA	NA	NA	6.40E-04
	PA	GxEprs_QT	$\hat{\alpha}_1$	-1.01E-01	5.01E-03	-2.02E+01	4.03E-90
			$\hat{\alpha}_2$	1.93E-01	3.27E-03	5.92E+01	0.00E+00
			$\hat{\alpha}_4$	2.71E-03	3.25E-03	8.35E-01	4.04E-01
			$\hat{\alpha}_3$	7.25E-03	5.00E-03	1.45E+00	1.47E-01
		GxEprs_QT*	$\hat{\alpha}_3$	NA	NA	NA	1.41E-01
	PALC	GxEprs_QT	$\hat{\alpha}_1$	2.28E-02	3.36E-03	6.77E+00	1.34E-11
			$\hat{\alpha}_2$	1.91E-01	3.21E-03	5.96E+01	0.00E+00
			$\hat{\alpha}_4$	2.52E-03	3.19E-03	7.88E-01	4.31E-01
			$\hat{\alpha}_3$	1.25E-02	3.16E-03	3.95E+00	7.72E-05
		GxEprs_QT*	$\hat{\alpha}_3$	NA	NA	NA	7.00E-05
	SMK	GxEprs_QT	$\hat{\alpha}_1$	5.91E-02	3.45E-03	1.71E+01	2.00E-65
			$\hat{\alpha}_2$	1.91E-01	3.32E-03	5.76E+01	0.00E+00

Continued on next page

Table S6 – *continued from previous page*

Phenotype	E	Model	Component	Estimate	Standard Error	Test Statistic	P value
			$\hat{\alpha_4}$	-4.26E-04	3.51E-03	-1.21E-01	9.04E-01
			$\hat{\alpha_3}$	1.17E-02	3.30E-03	3.54E+00	4.05E-04
		GxEprs_QT*	$\hat{\alpha_3}$	NA	NA	NA	3.20E-04
	NS	GxEprs_QT	$\hat{\alpha_1}$	1.68E-02	3.35E-03	5.02E+00	5.09E-07
			$\hat{\alpha_2}$	1.98E-01	3.32E-03	5.96E+01	0.00E+00
			$\hat{\alpha_4}$	-1.01E-02	3.31E-03	-3.04E+00	2.37E-03
			$\hat{\alpha_3}$	5.82E-03	3.28E-03	1.78E+00	7.58E-02
		GxEprs_QT*	$\hat{\alpha_3}$	NA	NA	NA	8.70E-02
WC	HD	GxEprs_QT	$\hat{\alpha_1}$	-6.95E-02	3.79E-03	-1.83E+01	8.28E-75
			$\hat{\alpha_2}$	2.26E-01	3.55E-03	6.38E+01	0.00E+00
			$\hat{\alpha_4}$	-3.59E-03	3.57E-03	-1.01E+00	3.14E-01
			$\hat{\alpha_3}$	1.22E-02	3.69E-03	3.31E+00	9.21E-04
		GxEprs_QT*	$\hat{\alpha_3}$	NA	NA	NA	1.25E-03
	PA	GxEprs_QT	$\hat{\alpha_1}$	-1.09E-01	5.31E-03	-2.06E+01	6.28E-94
			$\hat{\alpha_2}$	2.15E-01	3.85E-03	5.59E+01	0.00E+00
			$\hat{\alpha_4}$	2.86E-03	3.84E-03	7.43E-01	4.57E-01
			$\hat{\alpha_3}$	9.99E-03	5.29E-03	1.89E+00	5.91E-02
		GxEprs_QT*	$\hat{\alpha_3}$	NA	NA	NA	6.10E-02
	PALC	GxEprs_QT	$\hat{\alpha_1}$	3.96E-02	3.95E-03	1.00E+01	1.22E-23
			$\hat{\alpha_2}$	2.11E-01	3.75E-03	5.64E+01	0.00E+00
			$\hat{\alpha_4}$	2.30E-03	3.75E-03	6.13E-01	5.40E-01
			$\hat{\alpha_3}$	2.16E-02	3.72E-03	5.81E+00	6.31E-09
		GxEprs_QT*	$\hat{\alpha_3}$	NA	NA	NA	0.00E+00
	SMK	GxEprs_QT	$\hat{\alpha_1}$	1.06E-01	4.06E-03	2.61E+01	3.56E-149
			$\hat{\alpha_2}$	2.14E-01	3.89E-03	5.49E+01	0.00E+00
			$\hat{\alpha_4}$	3.87E-03	4.10E-03	9.45E-01	3.45E-01
			$\hat{\alpha_3}$	1.13E-02	3.88E-03	2.93E+00	3.41E-03
		GxEprs_QT*	$\hat{\alpha_3}$	NA	NA	NA	2.00E-03
	NS	GxEprs_QT	$\hat{\alpha_1}$	2.54E-02	4.01E-03	6.33E+00	2.48E-10
			$\hat{\alpha_2}$	2.21E-01	3.92E-03	5.63E+01	0.00E+00
			$\hat{\alpha_4}$	-4.48E-03	3.92E-03	-1.14E+00	2.54E-01
			$\hat{\alpha_3}$	9.22E-03	3.93E-03	2.35E+00	1.88E-02
		GxEprs_QT*	$\hat{\alpha_3}$	NA	NA	NA	2.80E-02

Table S7: Prediction accuracy and variance explained by GxE component for quantitative phenotypes across environmental variables

Phenotype	E	R^2 of GxEprs_QT	R^2 of GxEprs_QT_reduced	P value of ΔR^2	Var(GxE)
BMI	HD	1.0087E-01	1.0072E-01	4.2171E-03	4.4371E-03
	PA	1.0707E-01	1.0700E-01	6.3434E-02	3.0311E-03
	PALC	9.6913E-02	9.6380E-02	2.6993E-07	8.3641E-03
	SMK	9.7542E-02	9.7239E-02	1.5769E-04	6.3063E-03
	NS	9.5238E-02	9.5134E-02	2.7472E-02	3.6946E-03
WHR	HD	4.9284E-01	4.9280E-01	1.2410E-01	2.2913E-03
	PA	4.9973E-01	4.9972E-01	7.5069E-01	1.1457E-03
	PALC	4.9778E-01	4.9763E-01	5.7977E-03	4.4371E-03
	SMK	4.9454E-01	4.9450E-01	1.6919E-01	2.2913E-03
	NS	4.9114E-01	4.9110E-01	1.7290E-01	2.2913E-03
BF	HD	5.0353E-01	5.0343E-01	1.4568E-02	3.6229E-03
	PA	5.0804E-01	5.0802E-01	3.0892E-01	1.6202E-03
	PALC	4.9952E-01	4.9936E-01	5.1454E-03	4.5826E-03
	SMK	5.0314E-01	5.0301E-01	1.2623E-02	4.1307E-03
	NS	5.0141E-01	5.0138E-01	2.0981E-01	1.9843E-03
WC	HD	2.9336E-01	2.9322E-01	5.3296E-03	4.2867E-03
	PA	3.0819E-01	3.0814E-01	1.1629E-01	2.5618E-03
	PALC	3.0143E-01	3.0095E-01	1.1818E-06	7.9374E-03
	SMK	2.8894E-01	2.8881E-01	1.3512E-02	4.1307E-03
	NS	2.8784E-01	2.8775E-01	4.7374E-02	3.4370E-03

Table S8: Regression summary from the GxE PRS models across each outcome/environmental variable pair for binary traits

Phenotype	E	Model	Component	Estimate	Standard Error	Test Statistic	P value
OHCAN	BMI	GxEprs_BT	$\hat{\alpha}_1$	6.67E-02	2.27E-02	2.94E+00	3.25E-03
			$\hat{\alpha}_5$	-3.05E-02	2.34E-02	-1.31E+00	1.92E-01
			$\hat{\alpha}_2$	1.33E-01	1.86E-02	7.15E+00	8.97E-13
			$\hat{\alpha}_4$	1.30E-02	1.87E-02	6.97E-01	4.86E-01
			$\hat{\alpha}_3$	-3.98E-02	1.89E-02	-2.10E+00	3.58E-02
		GxEprs_BT*	$\hat{\alpha}_3$	NA	NA	NA	3.40E-02
	WHR	GxEprs_BT	$\hat{\alpha}_1$	8.29E-03	2.82E-02	2.94E-01	7.69E-01
			$\hat{\alpha}_5$	-2.24E-02	1.96E-02	-1.14E+00	2.53E-01
			$\hat{\alpha}_2$	1.32E-01	1.86E-02	7.07E+00	1.51E-12
			$\hat{\alpha}_4$	4.46E-02	1.88E-02	2.38E+00	1.75E-02
			$\hat{\alpha}_3$	4.16E-02	2.18E-02	1.90E+00	5.70E-02
		GxEprs_BT*	$\hat{\alpha}_3$	NA	NA	NA	6.10E-02
	HD	GxEprs_BT	$\hat{\alpha}_1$	-3.42E-02	2.00E-02	-1.70E+00	8.84E-02
			$\hat{\alpha}_5$	-1.97E-02	1.96E-02	-1.01E+00	3.14E-01
			$\hat{\alpha}_2$	1.34E-01	1.89E-02	7.11E+00	1.15E-12
			$\hat{\alpha}_4$	-2.87E-02	1.89E-02	-1.52E+00	1.28E-01
			$\hat{\alpha}_3$	-2.87E-03	1.90E-02	-1.51E-01	8.80E-01
		GxEprs_BT*	$\hat{\alpha}_3$	NA	NA	NA	8.29E-01
	NS	GxEprs_BT	$\hat{\alpha}_1$	1.62E-02	2.87E-02	5.64E-01	5.73E-01
			$\hat{\alpha}_5$	-1.10E-05	2.36E-02	-4.68E-04	1.00E+00
			$\hat{\alpha}_2$	1.20E-01	2.06E-02	5.85E+00	4.83E-09
			$\hat{\alpha}_4$	-1.23E-02	2.06E-02	-5.96E-01	5.51E-01
			$\hat{\alpha}_3$	4.09E-03	2.60E-02	1.57E-01	8.75E-01
		GxEprs_BT*	$\hat{\alpha}_3$	NA	NA	NA	8.23E-01
	BF	GxEprs_BT	$\hat{\alpha}_1$	6.01E-02	2.61E-02	2.31E+00	2.12E-02
			$\hat{\alpha}_5$	4.68E-02	1.84E-02	2.55E+00	1.08E-02
			$\hat{\alpha}_2$	1.29E-01	1.88E-02	6.86E+00	6.71E-12
			$\hat{\alpha}_4$	-3.89E-03	1.89E-02	-2.05E-01	8.37E-01
			$\hat{\alpha}_3$	2.91E-02	1.90E-02	1.53E+00	1.27E-01
		GxEprs_BT*	$\hat{\alpha}_3$	NA	NA	NA	1.09E-01
	PA	GxEprs_BT	$\hat{\alpha}_1$	-7.17E-02	4.16E-02	-1.72E+00	8.48E-02
			$\hat{\alpha}_5$	-1.72E-02	3.28E-02	-5.26E-01	5.99E-01
			$\hat{\alpha}_2$	1.24E-01	2.07E-02	6.02E+00	1.74E-09
			$\hat{\alpha}_4$	1.76E-02	2.07E-02	8.51E-01	3.95E-01
			$\hat{\alpha}_3$	-9.24E-02	3.47E-02	-2.66E+00	7.74E-03
		GxEprs_BT*	$\hat{\alpha}_3$	NA	NA	NA	8.00E-03
	WC	GxEprs_BT	$\hat{\alpha}_1$	1.05E-01	2.44E-02	4.29E+00	1.77E-05
			$\hat{\alpha}_5$	-9.97E-02	2.26E-02	-4.40E+00	1.06E-05
			$\hat{\alpha}_2$	1.32E-01	1.86E-02	7.09E+00	1.31E-12
			$\hat{\alpha}_4$	2.45E-02	1.87E-02	1.31E+00	1.91E-01
			$\hat{\alpha}_3$	1.62E-03	2.06E-02	7.85E-02	9.37E-01
		GxEprs_BT*	$\hat{\alpha}_3$	NA	NA	NA	9.85E-01
	PALC	GxEprs_BT	$\hat{\alpha}_1$	8.66E-02	4.38E-02	1.98E+00	4.79E-02
			$\hat{\alpha}_5$	-4.41E-02	3.44E-02	-1.28E+00	2.00E-01
			$\hat{\alpha}_2$	1.19E-01	2.00E-02	5.96E+00	2.53E-09
			$\hat{\alpha}_4$	1.67E-02	2.01E-02	8.29E-01	4.07E-01
			$\hat{\alpha}_3$	5.97E-02	3.82E-02	1.56E+00	1.18E-01
		GxEprs_BT*	$\hat{\alpha}_3$	NA	NA	NA	1.50E-01
	SMK	GxEprs_BT	$\hat{\alpha}_1$	-9.33E-03	4.77E-02	-1.95E-01	8.45E-01
			$\hat{\alpha}_5$	1.23E-02	3.26E-02	3.76E-01	7.07E-01
			$\hat{\alpha}_2$	1.04E-01	2.07E-02	5.01E+00	5.55E-07
			$\hat{\alpha}_4$	-1.46E-02	2.06E-02	-7.08E-01	4.79E-01
			$\hat{\alpha}_3$	2.85E-02	3.91E-02	7.29E-01	4.66E-01
		GxEprs_BT*	$\hat{\alpha}_3$	NA	NA	NA	4.14E-01
	BMI	GxEprs_BT	$\hat{\alpha}_1$	5.31E-01	1.41E-02	3.77E+01	0.00E+00
			$\hat{\alpha}_5$	-7.67E-02	1.25E-02	-6.15E+00	7.53E-10
			$\hat{\alpha}_2$	2.64E-01	1.13E-02	2.33E+01	1.97E-120
			$\hat{\alpha}_4$	4.34E-02	1.14E-02	3.80E+00	1.46E-04
			$\hat{\alpha}_3$	8.04E-03	1.08E-02	7.47E-01	4.55E-01
		GxEprs_BT*	$\hat{\alpha}_3$	NA	NA	NA	8.86E-01
	WHR	GxEprs_BT	$\hat{\alpha}_1$	5.22E-01	1.64E-02	3.18E+01	2.00E-222
			$\hat{\alpha}_5$	-1.47E-02	1.15E-02	-1.28E+00	2.01E-01
			$\hat{\alpha}_2$	2.62E-01	1.14E-02	2.30E+01	2.07E-117
			$\hat{\alpha}_4$	4.83E-02	1.15E-02	4.18E+00	2.88E-05
			$\hat{\alpha}_3$	2.56E-02	1.22E-02	2.09E+00	3.63E-02
		GxEprs_BT*	$\hat{\alpha}_3$	NA	NA	NA	2.10E-02
	HD	GxEprs_BT	$\hat{\alpha}_1$	-4.68E-02	1.31E-02	-3.58E+00	3.45E-04
			$\hat{\alpha}_5$	1.96E-02	1.10E-02	1.79E+00	7.40E-02
			$\hat{\alpha}_2$	2.62E-01	1.10E-02	2.38E+01	7.77E-125
			$\hat{\alpha}_4$	-7.45E-03	1.09E-02	-6.83E-01	4.95E-01
			$\hat{\alpha}_3$	-4.18E-03	1.24E-02	-3.37E-01	7.36E-01

Continued on next page

Table S8 – *continued from previous page*

Phenotype	E	Model	Component	Estimate	Standard Error	Test Statistic	P value
		GxEprs_BT*	$\hat{\alpha}_3$	NA	NA	NA	5.60E-01
	NS	GxEprs_BT	$\hat{\alpha}_1$	1.17E-01	1.42E-02	8.24E+00	1.67E-16
			$\hat{\alpha}_5$	7.13E-03	1.35E-02	5.28E-01	5.97E-01
			$\hat{\alpha}_2$	2.75E-01	1.22E-02	2.26E+01	1.49E-113
			$\hat{\alpha}_4$	1.98E-02	1.19E-02	1.66E+00	9.62E-02
			$\hat{\alpha}_3$	-1.22E-02	1.21E-02	-1.01E+00	3.14E-01
		GxEprs_BT*	$\hat{\alpha}_3$	NA	NA	NA	3.20E-01
	BF	GxEprs_BT	$\hat{\alpha}_1$	5.72E-01	1.63E-02	3.51E+01	4.51E-270
			$\hat{\alpha}_5$	1.39E-02	1.13E-02	1.23E+00	2.19E-01
			$\hat{\alpha}_2$	2.57E-01	1.12E-02	2.29E+01	1.82E-116
			$\hat{\alpha}_4$	2.44E-02	1.11E-02	2.19E+00	2.83E-02
			$\hat{\alpha}_3$	1.61E-02	1.11E-02	1.45E+00	1.48E-01
		GxEprs_BT*	$\hat{\alpha}_3$	NA	NA	NA	1.29E-01
	PA	GxEprs_BT	$\hat{\alpha}_1$	-1.46E-01	2.31E-02	-6.32E+00	2.67E-10
			$\hat{\alpha}_5$	8.42E-02	1.74E-02	4.83E+00	1.33E-06
			$\hat{\alpha}_2$	2.60E-01	1.22E-02	2.14E+01	2.08E-101
			$\hat{\alpha}_4$	-7.58E-03	1.20E-02	-6.30E-01	5.28E-01
			$\hat{\alpha}_3$	-7.00E-04	1.85E-02	-3.79E-02	9.70E-01
		GxEprs_BT*	$\hat{\alpha}_3$	NA	NA	NA	9.54E-01
	WC	GxEprs_BT	$\hat{\alpha}_1$	5.65E-01	1.51E-02	3.73E+01	4.20E-305
			$\hat{\alpha}_5$	-3.35E-02	1.18E-02	-2.84E+00	4.46E-03
			$\hat{\alpha}_2$	2.64E-01	1.15E-02	2.31E+01	9.64E-118
			$\hat{\alpha}_4$	5.27E-02	1.17E-02	4.52E+00	6.33E-06
			$\hat{\alpha}_3$	5.47E-03	1.19E-02	4.59E-01	6.46E-01
		GxEprs_BT*	$\hat{\alpha}_3$	NA	NA	NA	8.28E-01
	PALC	GxEprs_BT	$\hat{\alpha}_1$	1.10E-01	1.69E-02	6.51E+00	7.30E-11
			$\hat{\alpha}_5$	1.64E-02	1.51E-02	1.09E+00	2.77E-01
			$\hat{\alpha}_2$	2.39E-01	1.18E-02	2.02E+01	1.07E-90
			$\hat{\alpha}_4$	3.14E-02	1.17E-02	2.68E+00	7.44E-03
			$\hat{\alpha}_3$	1.35E-02	1.18E-02	1.15E+00	2.50E-01
		GxEprs_BT*	$\hat{\alpha}_3$	NA	NA	NA	2.84E-01
	SMK	GxEprs_BT	$\hat{\alpha}_1$	1.93E-01	1.86E-02	1.03E+01	4.55E-25
			$\hat{\alpha}_5$	-5.82E-02	1.69E-02	-3.43E+00	5.94E-04
			$\hat{\alpha}_2$	2.48E-01	1.23E-02	2.01E+01	9.46E-90
			$\hat{\alpha}_4$	3.22E-02	1.27E-02	2.55E+00	1.08E-02
			$\hat{\alpha}_3$	-5.57E-03	1.14E-02	-4.88E-01	6.26E-01
		GxEprs_BT*	$\hat{\alpha}_3$	NA	NA	NA	8.26E-01
CAD	BMI	GxEprs_BT	$\hat{\alpha}_1$	3.15E-01	2.58E-02	1.22E+01	2.92E-34
			$\hat{\alpha}_5$	-6.45E-02	1.97E-02	-3.27E+00	1.09E-03
			$\hat{\alpha}_2$	2.04E-01	1.77E-02	1.15E+01	1.15E-30
			$\hat{\alpha}_4$	3.10E-02	1.83E-02	1.69E+00	9.06E-02
			$\hat{\alpha}_3$	2.52E-02	2.13E-02	1.18E+00	2.37E-01
		GxEprs_BT*	$\hat{\alpha}_3$	NA	NA	NA	3.08E-01
	WHR	GxEprs_BT	$\hat{\alpha}_1$	3.57E-01	2.77E-02	1.29E+01	6.65E-38
			$\hat{\alpha}_5$	-5.76E-02	1.85E-02	-3.11E+00	1.88E-03
			$\hat{\alpha}_2$	2.20E-01	1.88E-02	1.17E+01	9.09E-32
			$\hat{\alpha}_4$	7.67E-02	2.05E-02	3.73E+00	1.88E-04
			$\hat{\alpha}_3$	9.52E-03	1.85E-02	5.15E-01	6.06E-01
		GxEprs_BT*	$\hat{\alpha}_3$	NA	NA	NA	5.24E-01
	HD	GxEprs_BT	$\hat{\alpha}_1$	-1.78E-02	1.96E-02	-9.10E-01	3.63E-01
			$\hat{\alpha}_5$	2.49E-02	1.71E-02	1.45E+00	1.47E-01
			$\hat{\alpha}_2$	2.11E-01	1.76E-02	1.20E+01	3.67E-33
			$\hat{\alpha}_4$	-2.55E-02	1.76E-02	-1.45E+00	1.48E-01
			$\hat{\alpha}_3$	-3.14E-02	1.84E-02	-1.71E+00	8.76E-02
		GxEprs_BT*	$\hat{\alpha}_3$	NA	NA	NA	1.14E-02
	NS	GxEprs_BT	$\hat{\alpha}_1$	1.17E-01	2.37E-02	4.93E+00	8.10E-07
			$\hat{\alpha}_5$	-8.95E-03	2.16E-02	-4.15E-01	6.78E-01
			$\hat{\alpha}_2$	1.86E-01	1.91E-02	9.70E+00	2.91E-22
			$\hat{\alpha}_4$	3.05E-02	1.91E-02	1.59E+00	1.11E-01
			$\hat{\alpha}_3$	2.76E-02	2.06E-02	1.34E+00	1.81E-01
		GxEprs_BT*	$\hat{\alpha}_3$	NA	NA	NA	1.53E-01
	BF	GxEprs_BT	$\hat{\alpha}_1$	3.23E-01	2.53E-02	1.27E+01	3.25E-37
			$\hat{\alpha}_5$	1.56E-02	1.80E-02	8.70E-01	3.84E-01
			$\hat{\alpha}_2$	2.04E-01	1.75E-02	1.17E+01	2.12E-31
			$\hat{\alpha}_4$	-9.37E-03	1.75E-02	-5.35E-01	5.92E-01
			$\hat{\alpha}_3$	1.96E-02	1.81E-02	1.09E+00	2.77E-01
		GxEprs_BT*	$\hat{\alpha}_3$	NA	NA	NA	1.65E-01
	PA	GxEprs_BT	$\hat{\alpha}_1$	-6.19E-02	4.31E-02	-1.44E+00	1.51E-01
			$\hat{\alpha}_5$	9.26E-02	2.49E-02	3.71E+00	2.04E-04
			$\hat{\alpha}_2$	2.09E-01	1.92E-02	1.09E+01	1.64E-27
			$\hat{\alpha}_4$	3.82E-02	1.93E-02	1.98E+00	4.76E-02
			$\hat{\alpha}_3$	3.65E-02	3.77E-02	9.68E-01	3.33E-01
		GxEprs_BT*	$\hat{\alpha}_3$	NA	NA	NA	4.33E-01
	WC	GxEprs_BT	$\hat{\alpha}_1$	3.19E-01	2.45E-02	1.30E+01	1.36E-38

Continued on next page

Table S8 – *continued from previous page*

Phenotype	E	Model	Component	Estimate	Standard Error	Test Statistic	P value
			$\hat{\alpha_5}$	-4.53E-02	1.80E-02	-2.51E+00	1.20E-02
			$\hat{\alpha_2}$	2.15E-01	1.84E-02	1.17E+01	2.13E-31
			$\hat{\alpha_4}$	5.50E-02	1.97E-02	2.79E+00	5.32E-03
			$\hat{\alpha_3}$	1.84E-02	1.75E-02	1.05E+00	2.95E-01
		GxEprs_BT*	$\hat{\alpha_3}$	NA	NA	NA	3.88E-01
	PALC	GxEprs_BT	$\hat{\alpha_1}$	-5.40E-03	2.60E-02	-2.07E-01	8.36E-01
			$\hat{\alpha_5}$	9.37E-03	2.07E-02	4.52E-01	6.52E-01
			$\hat{\alpha_2}$	2.06E-01	1.87E-02	1.10E+01	4.23E-28
			$\hat{\alpha_4}$	2.37E-03	1.87E-02	1.27E-01	8.99E-01
			$\hat{\alpha_3}$	5.17E-02	1.96E-02	2.63E+00	8.52E-03
		GxEprs_BT*	$\hat{\alpha_3}$	NA	NA	NA	4.00E-03
	SMK	GxEprs_BT	$\hat{\alpha_1}$	3.10E-01	2.73E-02	1.14E+01	5.11E-30
			$\hat{\alpha_5}$	-1.20E-01	2.35E-02	-5.09E+00	3.51E-07
			$\hat{\alpha_2}$	2.03E-01	1.97E-02	1.03E+01	6.72E-25
			$\hat{\alpha_4}$	2.66E-02	2.03E-02	1.31E+00	1.92E-01
			$\hat{\alpha_3}$	3.75E-03	1.53E-02	2.46E-01	8.06E-01
		GxEprs_BT*	$\hat{\alpha_3}$	NA	NA	NA	6.46E-01
DIAB	BMI	GxEprs_BT	$\hat{\alpha_1}$	9.20E-01	2.76E-02	3.33E+01	2.10E-243
			$\hat{\alpha_5}$	-1.65E-01	1.82E-02	-9.07E+00	1.21E-19
			$\hat{\alpha_2}$	3.39E-01	2.19E-02	1.55E+01	3.17E-54
			$\hat{\alpha_4}$	1.13E-01	2.47E-02	4.59E+00	4.40E-06
			$\hat{\alpha_3}$	3.60E-02	1.66E-02	2.17E+00	3.01E-02
		GxEprs_BT*	$\hat{\alpha_3}$	NA	NA	NA	5.90E-02
	WHR	GxEprs_BT	$\hat{\alpha_1}$	1.10E+00	3.59E-02	3.06E+01	4.43E-206
			$\hat{\alpha_5}$	-1.01E-01	1.94E-02	-5.21E+00	1.87E-07
			$\hat{\alpha_2}$	3.29E-01	2.24E-02	1.47E+01	5.08E-49
			$\hat{\alpha_4}$	1.82E-01	2.62E-02	6.94E+00	4.05E-12
			$\hat{\alpha_3}$	-4.69E-02	2.38E-02	-1.97E+00	4.84E-02
		GxEprs_BT*	$\hat{\alpha_3}$	NA	NA	NA	7.30E-02
	HD	GxEprs_BT	$\hat{\alpha_1}$	-9.91E-02	2.31E-02	-4.29E+00	1.75E-05
			$\hat{\alpha_5}$	2.10E-02	1.79E-02	1.17E+00	2.41E-01
			$\hat{\alpha_2}$	3.26E-01	1.88E-02	1.73E+01	2.03E-67
			$\hat{\alpha_4}$	2.82E-04	1.88E-02	1.50E-02	9.88E-01
			$\hat{\alpha_3}$	-3.88E-03	2.15E-02	-1.81E-01	8.57E-01
		GxEprs_BT*	$\hat{\alpha_3}$	NA	NA	NA	9.31E-01
	NS	GxEprs_BT	$\hat{\alpha_1}$	1.06E-01	2.58E-02	4.13E+00	3.63E-05
			$\hat{\alpha_5}$	3.12E-02	2.22E-02	1.41E+00	1.59E-01
			$\hat{\alpha_2}$	3.14E-01	2.04E-02	1.54E+01	2.01E-53
			$\hat{\alpha_4}$	3.26E-02	2.03E-02	1.61E+00	1.08E-01
			$\hat{\alpha_3}$	1.38E-02	2.24E-02	6.15E-01	5.38E-01
		GxEprs_BT*	$\hat{\alpha_3}$	NA	NA	NA	4.52E-01
	BF	GxEprs_BT	$\hat{\alpha_1}$	1.04E+00	3.06E-02	3.39E+01	8.87E-252
			$\hat{\alpha_5}$	8.80E-03	1.97E-02	4.46E-01	6.55E-01
			$\hat{\alpha_2}$	3.01E-01	1.95E-02	1.54E+01	8.77E-54
			$\hat{\alpha_4}$	5.33E-02	2.00E-02	2.66E+00	7.72E-03
			$\hat{\alpha_3}$	4.75E-02	1.90E-02	2.50E+00	1.24E-02
		GxEprs_BT*	$\hat{\alpha_3}$	NA	NA	NA	1.10E-02
	PA	GxEprs_BT	$\hat{\alpha_1}$	-2.91E-01	4.31E-02	-6.77E+00	1.32E-11
			$\hat{\alpha_5}$	1.63E-01	2.76E-02	5.91E+00	3.48E-09
			$\hat{\alpha_2}$	2.91E-01	2.08E-02	1.40E+01	3.13E-44
			$\hat{\alpha_4}$	-7.14E-03	2.06E-02	-3.46E-01	7.29E-01
			$\hat{\alpha_3}$	-3.12E-02	3.75E-02	-8.31E-01	4.06E-01
		GxEprs_BT*	$\hat{\alpha_3}$	NA	NA	NA	2.99E-01
	WC	GxEprs_BT	$\hat{\alpha_1}$	1.07E+00	3.41E-02	3.15E+01	2.47E-217
			$\hat{\alpha_5}$	-1.27E-01	1.87E-02	-6.79E+00	1.10E-11
			$\hat{\alpha_2}$	3.49E-01	2.31E-02	1.51E+01	1.48E-51
			$\hat{\alpha_4}$	1.76E-01	2.75E-02	6.42E+00	1.40E-10
			$\hat{\alpha_3}$	-2.29E-03	2.22E-02	-1.03E-01	9.18E-01
		GxEprs_BT*	$\hat{\alpha_3}$	NA	NA	NA	9.58E-01
	PALC	GxEprs_BT	$\hat{\alpha_1}$	-1.72E-01	3.75E-02	-4.58E+00	4.67E-06
			$\hat{\alpha_5}$	9.58E-02	1.87E-02	5.13E+00	2.85E-07
			$\hat{\alpha_2}$	3.00E-01	2.02E-02	1.49E+01	6.33E-50
			$\hat{\alpha_4}$	-2.44E-02	2.00E-02	-1.22E+00	2.23E-01
			$\hat{\alpha_3}$	-4.67E-02	3.05E-02	-1.53E+00	1.25E-01
		GxEprs_BT*	$\hat{\alpha_3}$	NA	NA	NA	1.64E-01
	SMK	GxEprs_BT	$\hat{\alpha_1}$	2.80E-01	2.91E-02	9.62E+00	6.59E-22
			$\hat{\alpha_5}$	-6.52E-02	2.20E-02	-2.96E+00	3.03E-03
			$\hat{\alpha_2}$	3.12E-01	2.14E-02	1.46E+01	2.93E-48
			$\hat{\alpha_4}$	8.78E-02	2.22E-02	3.95E+00	7.69E-05
			$\hat{\alpha_3}$	-6.12E-03	1.80E-02	-3.41E-01	7.33E-01
		GxEprs_BT*	$\hat{\alpha_3}$	NA	NA	NA	5.70E-01
	BMI	GxEprs_BT	$\hat{\alpha_1}$	1.19E-01	1.73E-02	6.89E+00	5.39E-12
			$\hat{\alpha_5}$	-3.40E-02	1.62E-02	-2.09E+00	3.63E-02
			$\hat{\alpha_2}$	1.44E-01	1.37E-02	1.05E+01	9.70E-26

Continued on next page

Table S8 – *continued from previous page*

Phenotype	E	Model	Component	Estimate	Standard Error	Test Statistic	P value
			$\hat{\alpha}_4$	3.71E-02	1.37E-02	2.70E+00	6.93E-03
			$\hat{\alpha}_3$	-1.23E-03	1.41E-02	-8.66E-02	9.31E-01
		GxEprs_BT*	$\hat{\alpha}_3$	NA	NA	NA	9.74E-01
	WHR	GxEprs_BT	$\hat{\alpha}_1$	1.90E-01	2.11E-02	9.01E+00	2.01E-19
			$\hat{\alpha}_5$	-9.26E-02	1.50E-02	-6.18E+00	6.35E-10
			$\hat{\alpha}_2$	1.45E-01	1.39E-02	1.04E+01	1.76E-25
			$\hat{\alpha}_4$	2.84E-02	1.43E-02	1.99E+00	4.66E-02
			$\hat{\alpha}_3$	2.56E-02	1.57E-02	1.63E+00	1.02E-01
		GxEprs_BT*	$\hat{\alpha}_3$	NA	NA	NA	1.06E-01
	HD	GxEprs_BT	$\hat{\alpha}_1$	-5.68E-02	1.50E-02	-3.80E+00	1.45E-04
			$\hat{\alpha}_5$	-5.27E-03	1.39E-02	-3.80E-01	7.04E-01
			$\hat{\alpha}_2$	1.41E-01	1.39E-02	1.02E+01	2.66E-24
			$\hat{\alpha}_4$	2.67E-02	1.40E-02	1.91E+00	5.64E-02
			$\hat{\alpha}_3$	-2.43E-03	1.38E-02	-1.76E-01	8.60E-01
		GxEprs_BT*	$\hat{\alpha}_3$	NA	NA	NA	8.39E-01
	NS	GxEprs_BT	$\hat{\alpha}_1$	1.15E-01	1.89E-02	6.07E+00	1.30E-09
			$\hat{\alpha}_5$	-4.97E-03	1.69E-02	-2.94E-01	7.69E-01
			$\hat{\alpha}_2$	1.31E-01	1.52E-02	8.65E+00	5.31E-18
			$\hat{\alpha}_4$	-7.88E-03	1.52E-02	-5.19E-01	6.04E-01
			$\hat{\alpha}_3$	-5.17E-02	1.64E-02	-3.16E+00	1.59E-03
		GxEprs_BT*	$\hat{\alpha}_3$	NA	NA	NA	8.00E-03
	BF	GxEprs_BT	$\hat{\alpha}_1$	1.16E-01	2.05E-02	5.67E+00	1.43E-08
			$\hat{\alpha}_5$	9.88E-02	1.28E-02	7.69E+00	1.46E-14
			$\hat{\alpha}_2$	1.41E-01	1.37E-02	1.03E+01	9.31E-25
			$\hat{\alpha}_4$	6.76E-03	1.37E-02	4.93E-01	6.22E-01
			$\hat{\alpha}_3$	1.58E-02	1.58E-02	1.00E+00	3.16E-01
		GxEprs_BT*	$\hat{\alpha}_3$	NA	NA	NA	3.17E-01
	PA	GxEprs_BT	$\hat{\alpha}_1$	-3.91E-02	2.71E-02	-1.44E+00	1.49E-01
			$\hat{\alpha}_5$	3.75E-02	2.10E-02	1.78E+00	7.49E-02
			$\hat{\alpha}_2$	1.34E-01	1.52E-02	8.82E+00	1.17E-18
			$\hat{\alpha}_4$	2.30E-02	1.52E-02	1.52E+00	1.29E-01
			$\hat{\alpha}_3$	-1.47E-02	2.11E-02	-6.97E-01	4.86E-01
		GxEprs_BT*	$\hat{\alpha}_3$	NA	NA	NA	5.48E-01
	WC	GxEprs_BT	$\hat{\alpha}_1$	1.87E-01	1.85E-02	1.01E+01	6.76E-24
			$\hat{\alpha}_5$	-1.18E-01	1.60E-02	-7.37E+00	1.73E-13
			$\hat{\alpha}_2$	1.45E-01	1.39E-02	1.05E+01	1.15E-25
			$\hat{\alpha}_4$	4.27E-02	1.41E-02	3.02E+00	2.49E-03
			$\hat{\alpha}_3$	1.28E-02	1.47E-02	8.66E-01	3.87E-01
		GxEprs_BT*	$\hat{\alpha}_3$	NA	NA	NA	3.40E-01
	PALC	GxEprs_BT	$\hat{\alpha}_1$	-9.74E-02	2.88E-02	-3.39E+00	7.09E-04
			$\hat{\alpha}_5$	6.42E-02	1.56E-02	4.10E+00	4.05E-05
			$\hat{\alpha}_2$	1.28E-01	1.48E-02	8.67E+00	4.34E-18
			$\hat{\alpha}_4$	7.33E-03	1.48E-02	4.96E-01	6.20E-01
			$\hat{\alpha}_3$	-1.37E-02	2.51E-02	-5.45E-01	5.86E-01
		GxEprs_BT*	$\hat{\alpha}_3$	NA	NA	NA	6.02E-01
	SMK	GxEprs_BT	$\hat{\alpha}_1$	7.37E-02	2.39E-02	3.09E+00	2.00E-03
			$\hat{\alpha}_5$	-4.01E-02	2.19E-02	-1.83E+00	6.72E-02
			$\hat{\alpha}_2$	1.19E-01	1.53E-02	7.81E+00	5.72E-15
			$\hat{\alpha}_4$	6.34E-03	1.54E-02	4.12E-01	6.80E-01
			$\hat{\alpha}_3$	-8.55E-03	1.43E-02	-5.98E-01	5.50E-01
		GxEprs_BT*	$\hat{\alpha}_3$	NA	NA	NA	5.29E-01
	BMI	GxEprs_BT	$\hat{\alpha}_1$	2.50E-01	2.29E-02	1.09E+01	1.21E-27
			$\hat{\alpha}_5$	-7.78E-03	1.71E-02	-4.56E-01	6.48E-01
			$\hat{\alpha}_2$	1.41E-01	1.98E-02	7.14E+00	9.18E-13
			$\hat{\alpha}_4$	1.08E-03	2.03E-02	5.32E-02	9.58E-01
			$\hat{\alpha}_3$	1.08E-02	1.68E-02	6.43E-01	5.21E-01
		GxEprs_BT*	$\hat{\alpha}_3$	NA	NA	NA	4.44E-01
	WHR	GxEprs_BT	$\hat{\alpha}_1$	4.03E-01	2.62E-02	1.54E+01	2.27E-53
			$\hat{\alpha}_5$	-2.15E-02	1.81E-02	-1.19E+00	2.35E-01
			$\hat{\alpha}_2$	1.37E-01	1.94E-02	7.05E+00	1.79E-12
			$\hat{\alpha}_4$	1.16E-02	1.95E-02	5.95E-01	5.52E-01
			$\hat{\alpha}_3$	3.16E-02	1.96E-02	1.62E+00	1.06E-01
		GxEprs_BT*	$\hat{\alpha}_3$	NA	NA	NA	1.11E-01
	HD	GxEprs_BT	$\hat{\alpha}_1$	-8.30E-02	2.30E-02	-3.61E+00	3.06E-04
			$\hat{\alpha}_5$	5.18E-03	1.97E-02	2.63E-01	7.93E-01
			$\hat{\alpha}_2$	1.36E-01	1.98E-02	6.89E+00	5.75E-12
			$\hat{\alpha}_4$	4.07E-02	1.98E-02	2.06E+00	3.95E-02
			$\hat{\alpha}_3$	-4.37E-03	2.14E-02	-2.04E-01	8.38E-01
		GxEprs_BT*	$\hat{\alpha}_3$	NA	NA	NA	9.28E-01
	NS	GxEprs_BT	$\hat{\alpha}_1$	8.02E-01	3.98E-02	2.02E+01	2.28E-90
			$\hat{\alpha}_5$	-3.57E-02	2.41E-02	-1.48E+00	1.39E-01
			$\hat{\alpha}_2$	1.13E-01	2.69E-02	4.18E+00	2.91E-05
			$\hat{\alpha}_4$	6.46E-02	3.16E-02	2.04E+00	4.10E-02
			$\hat{\alpha}_3$	2.01E-02	2.84E-02	7.07E-01	4.80E-01

Continued on next page

Table S8 – *continued from previous page*

Phenotype	E	Model	Component	Estimate	Standard Error	Test Statistic	P value
		GxEprs_BT*	$\hat{\alpha_3}$	NA	NA	NA	4.14E-01
	BF	GxEprs_BT	$\hat{\alpha_1}$	2.99E-01	2.84E-02	1.05E+01	7.51E-26
			$\hat{\alpha_5}$	5.34E-02	1.88E-02	2.84E+00	4.45E-03
			$\hat{\alpha_2}$	1.41E-01	2.02E-02	6.99E+00	2.74E-12
			$\hat{\alpha_4}$	5.33E-03	2.10E-02	2.54E-01	8.00E-01
			$\hat{\alpha_3}$	1.66E-02	1.98E-02	8.37E-01	4.02E-01
		GxEprs_BT*	$\hat{\alpha_3}$	NA	NA	NA	4.08E-01
	PA	GxEprs_BT	$\hat{\alpha_1}$	-1.26E-01	4.26E-02	-2.96E+00	3.03E-03
			$\hat{\alpha_5}$	8.46E-02	3.12E-02	2.72E+00	6.62E-03
			$\hat{\alpha_2}$	1.36E-01	2.20E-02	6.17E+00	6.92E-10
			$\hat{\alpha_4}$	-2.45E-03	2.20E-02	-1.12E-01	9.11E-01
			$\hat{\alpha_3}$	6.28E-03	3.57E-02	1.76E-01	8.60E-01
		GxEprs_BT*	$\hat{\alpha_3}$	NA	NA	NA	8.90E-01
	WC	GxEprs_BT	$\hat{\alpha_1}$	3.48E-01	2.36E-02	1.48E+01	2.43E-49
			$\hat{\alpha_5}$	-2.40E-02	1.76E-02	-1.36E+00	1.73E-01
			$\hat{\alpha_2}$	1.39E-01	1.97E-02	7.04E+00	1.89E-12
			$\hat{\alpha_4}$	8.46E-03	2.00E-02	4.23E-01	6.72E-01
			$\hat{\alpha_3}$	6.78E-03	1.84E-02	3.68E-01	7.13E-01
		GxEprs_BT*	$\hat{\alpha_3}$	NA	NA	NA	7.19E-01
	PALC	GxEprs_BT	$\hat{\alpha_1}$	3.37E-03	4.99E-02	6.76E-02	9.46E-01
			$\hat{\alpha_5}$	9.51E-02	1.92E-02	4.95E+00	7.29E-07
			$\hat{\alpha_2}$	1.28E-01	2.13E-02	6.01E+00	1.89E-09
			$\hat{\alpha_4}$	3.34E-02	2.13E-02	1.57E+00	1.17E-01
			$\hat{\alpha_3}$	9.35E-02	4.69E-02	1.99E+00	4.62E-02
		GxEprs_BT*	$\hat{\alpha_3}$	NA	NA	NA	8.10E-02
	SMK	GxEprs_BT	$\hat{\alpha_1}$	3.46E-01	4.30E-02	8.05E+00	7.97E-16
			$\hat{\alpha_5}$	-7.52E-02	2.76E-02	-2.72E+00	6.47E-03
			$\hat{\alpha_2}$	1.16E-01	2.25E-02	5.18E+00	2.22E-07
			$\hat{\alpha_4}$	1.77E-03	2.29E-02	7.74E-02	9.38E-01
			$\hat{\alpha_3}$	9.66E-02	3.42E-02	2.82E+00	4.78E-03
		GxEprs_BT*	$\hat{\alpha_3}$	NA	NA	NA	8.00E-03
CATA	BMI	GxEprs_BT	$\hat{\alpha_1}$	4.55E-02	1.79E-02	2.54E+00	1.11E-02
			$\hat{\alpha_5}$	3.50E-02	1.61E-02	2.17E+00	3.01E-02
			$\hat{\alpha_2}$	1.59E-01	1.46E-02	1.09E+01	9.69E-28
			$\hat{\alpha_4}$	3.05E-03	1.46E-02	2.09E-01	8.35E-01
			$\hat{\alpha_3}$	-2.24E-02	1.49E-02	-1.51E+00	1.32E-01
		GxEprs_BT*	$\hat{\alpha_3}$	NA	NA	NA	1.19E-01
	WHR	GxEprs_BT	$\hat{\alpha_1}$	8.17E-02	1.99E-02	4.11E+00	4.03E-05
			$\hat{\alpha_5}$	5.47E-02	1.36E-02	4.02E+00	5.75E-05
			$\hat{\alpha_2}$	1.57E-01	1.46E-02	1.08E+01	3.02E-27
			$\hat{\alpha_4}$	1.79E-02	1.46E-02	1.22E+00	2.21E-01
			$\hat{\alpha_3}$	3.74E-02	1.49E-02	2.51E+00	1.22E-02
		GxEprs_BT*	$\hat{\alpha_3}$	NA	NA	NA	1.90E-02
	HD	GxEprs_BT	$\hat{\alpha_1}$	5.71E-03	1.58E-02	3.62E-01	7.17E-01
			$\hat{\alpha_5}$	1.50E-02	1.51E-02	9.93E-01	3.21E-01
			$\hat{\alpha_2}$	1.61E-01	1.48E-02	1.09E+01	1.09E-27
			$\hat{\alpha_4}$	2.75E-02	1.48E-02	1.86E+00	6.29E-02
			$\hat{\alpha_3}$	-1.93E-03	1.52E-02	-1.27E-01	8.99E-01
		GxEprs_BT*	$\hat{\alpha_3}$	NA	NA	NA	8.47E-01
	NS	GxEprs_BT	$\hat{\alpha_1}$	9.66E-02	2.88E-02	3.36E+00	7.77E-04
			$\hat{\alpha_5}$	-7.72E-03	1.86E-02	-4.16E-01	6.77E-01
			$\hat{\alpha_2}$	1.55E-01	1.63E-02	9.52E+00	1.69E-21
			$\hat{\alpha_4}$	-9.79E-03	1.63E-02	-6.01E-01	5.48E-01
			$\hat{\alpha_3}$	1.22E-02	2.69E-02	4.52E-01	6.51E-01
		GxEprs_BT*	$\hat{\alpha_3}$	NA	NA	NA	6.28E-01
	BF	GxEprs_BT	$\hat{\alpha_1}$	8.38E-02	2.11E-02	3.97E+00	7.10E-05
			$\hat{\alpha_5}$	2.66E-02	1.50E-02	1.78E+00	7.57E-02
			$\hat{\alpha_2}$	1.60E-01	1.48E-02	1.08E+01	4.04E-27
			$\hat{\alpha_4}$	-1.06E-02	1.50E-02	-7.10E-01	4.78E-01
			$\hat{\alpha_3}$	-1.14E-02	1.53E-02	-7.41E-01	4.59E-01
		GxEprs_BT*	$\hat{\alpha_3}$	NA	NA	NA	8.31E-01
	PA	GxEprs_BT	$\hat{\alpha_1}$	-8.83E-02	3.41E-02	-2.59E+00	9.57E-03
			$\hat{\alpha_5}$	4.07E-02	2.46E-02	1.65E+00	9.90E-02
			$\hat{\alpha_2}$	1.79E-01	1.64E-02	1.09E+01	1.23E-27
			$\hat{\alpha_4}$	9.06E-03	1.63E-02	5.55E-01	5.79E-01
			$\hat{\alpha_3}$	-2.09E-02	2.97E-02	-7.06E-01	4.80E-01
		GxEprs_BT*	$\hat{\alpha_3}$	NA	NA	NA	7.23E-01
	WC	GxEprs_BT	$\hat{\alpha_1}$	7.33E-02	1.74E-02	4.20E+00	2.62E-05
			$\hat{\alpha_5}$	6.29E-02	1.44E-02	4.37E+00	1.25E-05
			$\hat{\alpha_2}$	1.57E-01	1.46E-02	1.08E+01	5.55E-27
			$\hat{\alpha_4}$	2.07E-03	1.46E-02	1.41E-01	8.88E-01
			$\hat{\alpha_3}$	5.74E-03	1.42E-02	4.04E-01	6.86E-01
		GxEprs_BT*	$\hat{\alpha_3}$	NA	NA	NA	9.65E-01
	PALC	GxEprs_BT	$\hat{\alpha_1}$	2.32E-02	2.24E-02	1.04E+00	2.99E-01

Continued on next page

Table S8 – *continued from previous page*

Phenotype	E	Model	Component	Estimate	Standard Error	Test Statistic	P value
			$\hat{\alpha_5}$	4.20E-02	1.71E-02	2.46E+00	1.38E-02
			$\hat{\alpha_2}$	1.57E-01	1.58E-02	9.94E+00	2.77E-23
			$\hat{\alpha_4}$	1.85E-02	1.57E-02	1.18E+00	2.38E-01
			$\hat{\alpha_3}$	2.72E-02	1.66E-02	1.64E+00	1.01E-01
		GxEprs_BT*	$\hat{\alpha_3}$	NA	NA	NA	1.02E-01
	SMK	GxEprs_BT	$\hat{\alpha_1}$	1.47E-01	2.43E-02	6.04E+00	1.54E-09
			$\hat{\alpha_5}$	-5.10E-02	2.21E-02	-2.31E+00	2.09E-02
			$\hat{\alpha_2}$	1.38E-01	1.63E-02	8.43E+00	3.37E-17
			$\hat{\alpha_4}$	6.60E-03	1.65E-02	4.01E-01	6.88E-01
			$\hat{\alpha_3}$	1.90E-02	1.37E-02	1.39E+00	1.65E-01
		GxEprs_BT*	$\hat{\alpha_3}$	NA	NA	NA	1.79E-01
STRO	BMI	GxEprs_BT	$\hat{\alpha_1}$	-1.68E-02	6.39E-02	-2.63E-01	7.92E-01
			$\hat{\alpha_5}$	-6.63E-02	4.05E-02	-1.64E+00	1.02E-01
			$\hat{\alpha_2}$	2.98E-02	2.97E-02	1.00E+00	3.17E-01
			$\hat{\alpha_4}$	-2.17E-02	3.02E-02	-7.17E-01	4.73E-01
			$\hat{\alpha_3}$	-1.36E-01	5.95E-02	-2.28E+00	2.26E-02
		GxEprs_BT*	$\hat{\alpha_3}$	NA	NA	NA	1.40E-02
	WHR	GxEprs_BT	$\hat{\alpha_1}$	8.99E-02	4.69E-02	1.92E+00	5.52E-02
			$\hat{\alpha_5}$	-6.66E-02	3.23E-02	-2.06E+00	3.96E-02
			$\hat{\alpha_2}$	3.26E-02	3.08E-02	1.06E+00	2.90E-01
			$\hat{\alpha_4}$	3.31E-02	3.23E-02	1.02E+00	3.06E-01
			$\hat{\alpha_3}$	-1.21E-01	3.55E-02	-3.40E+00	6.62E-04
		GxEprs_BT*	$\hat{\alpha_3}$	NA	NA	NA	4.00E-04
	HD	GxEprs_BT	$\hat{\alpha_1}$	4.97E-02	5.26E-02	9.45E-01	3.45E-01
			$\hat{\alpha_5}$	-8.18E-04	3.02E-02	-2.71E-02	9.78E-01
			$\hat{\alpha_2}$	3.92E-02	3.00E-02	1.31E+00	1.91E-01
			$\hat{\alpha_4}$	-7.11E-03	3.02E-02	-2.36E-01	8.14E-01
			$\hat{\alpha_3}$	-9.17E-02	5.15E-02	-1.78E+00	7.50E-02
		GxEprs_BT*	$\hat{\alpha_3}$	NA	NA	NA	9.60E-02
	NS	GxEprs_BT	$\hat{\alpha_1}$	-1.44E-02	3.77E-02	-3.82E-01	7.02E-01
			$\hat{\alpha_5}$	7.38E-02	3.72E-02	1.98E+00	4.73E-02
			$\hat{\alpha_2}$	1.27E-02	3.30E-02	3.85E-01	7.00E-01
			$\hat{\alpha_4}$	1.95E-02	3.32E-02	5.87E-01	5.57E-01
			$\hat{\alpha_3}$	2.11E-02	3.27E-02	6.45E-01	5.19E-01
		GxEprs_BT*	$\hat{\alpha_3}$	NA	NA	NA	4.93E-01
	BF	GxEprs_BT	$\hat{\alpha_1}$	5.86E-02	4.26E-02	1.38E+00	1.68E-01
			$\hat{\alpha_5}$	5.77E-02	2.91E-02	1.98E+00	4.72E-02
			$\hat{\alpha_2}$	3.30E-02	2.96E-02	1.12E+00	2.65E-01
			$\hat{\alpha_4}$	-7.56E-03	2.98E-02	-2.54E-01	8.00E-01
			$\hat{\alpha_3}$	-3.79E-02	3.13E-02	-1.21E+00	2.26E-01
		GxEprs_BT*	$\hat{\alpha_3}$	NA	NA	NA	1.63E-01
	PA	GxEprs_BT	$\hat{\alpha_1}$	6.13E-02	1.11E-01	5.54E-01	5.80E-01
			$\hat{\alpha_5}$	5.30E-04	4.90E-02	1.08E-02	9.91E-01
			$\hat{\alpha_2}$	2.26E-02	3.27E-02	6.92E-01	4.89E-01
			$\hat{\alpha_4}$	3.41E-02	3.30E-02	1.03E+00	3.02E-01
			$\hat{\alpha_3}$	8.28E-02	1.05E-01	7.91E-01	4.29E-01
		GxEprs_BT*	$\hat{\alpha_3}$	NA	NA	NA	4.41E-01
	WC	GxEprs_BT	$\hat{\alpha_1}$	6.59E-02	5.05E-02	1.30E+00	1.92E-01
			$\hat{\alpha_5}$	-7.76E-02	3.45E-02	-2.25E+00	2.45E-02
			$\hat{\alpha_2}$	3.43E-02	3.04E-02	1.13E+00	2.59E-01
			$\hat{\alpha_4}$	-7.20E-03	3.15E-02	-2.28E-01	8.19E-01
			$\hat{\alpha_3}$	-1.16E-01	4.37E-02	-2.65E+00	8.05E-03
		GxEprs_BT*	$\hat{\alpha_3}$	NA	NA	NA	8.00E-03
	PALC	GxEprs_BT	$\hat{\alpha_1}$	-1.62E-01	8.77E-02	-1.84E+00	6.51E-02
			$\hat{\alpha_5}$	-3.05E-02	3.71E-02	-8.23E-01	4.11E-01
			$\hat{\alpha_2}$	1.03E-02	3.18E-02	3.25E-01	7.45E-01
			$\hat{\alpha_4}$	1.93E-03	3.19E-02	6.06E-02	9.52E-01
			$\hat{\alpha_3}$	-2.34E-01	8.36E-02	-2.80E+00	5.08E-03
		GxEprs_BT*	$\hat{\alpha_3}$	NA	NA	NA	7.00E-03
	SMK	GxEprs_BT	$\hat{\alpha_1}$	2.16E-01	8.00E-02	2.69E+00	7.04E-03
			$\hat{\alpha_5}$	-1.57E-01	4.84E-02	-3.25E+00	1.15E-03
			$\hat{\alpha_2}$	6.49E-02	3.37E-02	1.93E+00	5.40E-02
			$\hat{\alpha_4}$	-2.11E-02	3.47E-02	-6.08E-01	5.43E-01
			$\hat{\alpha_3}$	-6.03E-02	6.77E-02	-8.91E-01	3.73E-01
		GxEprs_BT*	$\hat{\alpha_3}$	NA	NA	NA	3.65E-01
	BMI	GxEprs_BT	$\hat{\alpha_1}$	2.31E-01	2.40E-02	9.65E+00	4.83E-22
			$\hat{\alpha_5}$	-1.93E-02	1.88E-02	-1.02E+00	3.06E-01
			$\hat{\alpha_2}$	3.03E-01	1.99E-02	1.52E+01	3.03E-52
			$\hat{\alpha_4}$	2.01E-02	2.01E-02	9.97E-01	3.19E-01
			$\hat{\alpha_3}$	-3.19E-02	1.88E-02	-1.69E+00	9.04E-02
		GxEprs_BT*	$\hat{\alpha_3}$	NA	NA	NA	1.28E-01
	WHR	GxEprs_BT	$\hat{\alpha_1}$	1.76E-01	2.61E-02	6.74E+00	1.57E-11
			$\hat{\alpha_5}$	-1.20E-02	2.07E-02	-5.79E-01	5.62E-01
			$\hat{\alpha_2}$	3.05E-01	1.98E-02	1.54E+01	9.66E-54

Continued on next page

Table S8 – *continued from previous page*

Phenotype	E	Model	Component	Estimate	Standard Error	Test Statistic	P value
			$\hat{\alpha}_4$	-3.84E-02	1.99E-02	-1.93E+00	5.40E-02
			$\hat{\alpha}_3$	1.12E-02	2.08E-02	5.39E-01	5.90E-01
		GxEprs_BT*	$\hat{\alpha}_3$	NA	NA	NA	4.88E-01
	HD	GxEprs_BT	$\hat{\alpha}_1$	1.21E-02	2.14E-02	5.68E-01	5.70E-01
			$\hat{\alpha}_5$	1.84E-02	2.08E-02	8.85E-01	3.76E-01
			$\hat{\alpha}_2$	3.18E-01	2.01E-02	1.58E+01	1.98E-56
			$\hat{\alpha}_4$	3.97E-02	2.02E-02	1.97E+00	4.94E-02
			$\hat{\alpha}_3$	-3.01E-03	2.08E-02	-1.45E-01	8.85E-01
		GxEprs_BT*	$\hat{\alpha}_3$	NA	NA	NA	7.34E-01
	NS	GxEprs_BT	$\hat{\alpha}_1$	1.24E-01	2.87E-02	4.31E+00	1.67E-05
			$\hat{\alpha}_5$	-2.45E-02	2.46E-02	-9.96E-01	3.19E-01
			$\hat{\alpha}_2$	2.71E-01	2.22E-02	1.22E+01	2.63E-34
			$\hat{\alpha}_4$	4.76E-02	2.24E-02	2.13E+00	3.34E-02
			$\hat{\alpha}_3$	-2.84E-02	2.42E-02	-1.17E+00	2.41E-01
		GxEprs_BT*	$\hat{\alpha}_3$	NA	NA	NA	2.09E-01
	BF	GxEprs_BT	$\hat{\alpha}_1$	2.34E-01	3.32E-02	7.06E+00	1.68E-12
			$\hat{\alpha}_5$	4.51E-02	2.09E-02	2.16E+00	3.12E-02
			$\hat{\alpha}_2$	3.32E-01	2.17E-02	1.53E+01	1.14E-52
			$\hat{\alpha}_4$	1.15E-01	2.38E-02	4.84E+00	1.29E-06
			$\hat{\alpha}_3$	-6.21E-03	2.43E-02	-2.55E-01	7.99E-01
		GxEprs_BT*	$\hat{\alpha}_3$	NA	NA	NA	7.80E-01
	PA	GxEprs_BT	$\hat{\alpha}_1$	-1.04E-01	5.26E-02	-1.97E+00	4.83E-02
			$\hat{\alpha}_5$	8.03E-02	3.36E-02	2.39E+00	1.69E-02
			$\hat{\alpha}_2$	2.72E-01	2.21E-02	1.23E+01	8.84E-35
			$\hat{\alpha}_4$	-3.86E-03	2.21E-02	-1.75E-01	8.61E-01
			$\hat{\alpha}_3$	1.30E-02	4.64E-02	2.80E-01	7.80E-01
		GxEprs_BT*	$\hat{\alpha}_3$	NA	NA	NA	9.21E-01
	WC	GxEprs_BT	$\hat{\alpha}_1$	2.60E-01	2.33E-02	1.12E+01	5.58E-29
			$\hat{\alpha}_5$	-2.14E-02	1.95E-02	-1.09E+00	2.74E-01
			$\hat{\alpha}_2$	2.97E-01	1.96E-02	1.51E+01	1.03E-51
			$\hat{\alpha}_4$	-1.29E-02	1.95E-02	-6.62E-01	5.08E-01
			$\hat{\alpha}_3$	-2.34E-02	2.01E-02	-1.16E+00	2.46E-01
		GxEprs_BT*	$\hat{\alpha}_3$	NA	NA	NA	3.55E-01
	PALC	GxEprs_BT	$\hat{\alpha}_1$	-1.99E-01	7.16E-02	-2.79E+00	5.34E-03
			$\hat{\alpha}_5$	1.14E-01	2.28E-02	5.03E+00	4.89E-07
			$\hat{\alpha}_2$	3.08E-01	2.27E-02	1.36E+01	5.80E-42
			$\hat{\alpha}_4$	-1.17E-01	2.37E-02	-4.93E+00	8.24E-07
			$\hat{\alpha}_3$	4.23E-02	6.81E-02	6.21E-01	5.34E-01
		GxEprs_BT*	$\hat{\alpha}_3$	NA	NA	NA	5.47E-01
	SMK	GxEprs_BT	$\hat{\alpha}_1$	1.48E-01	5.59E-02	2.64E+00	8.24E-03
			$\hat{\alpha}_5$	-1.08E-01	4.23E-02	-2.54E+00	1.09E-02
			$\hat{\alpha}_2$	3.06E-01	2.17E-02	1.41E+01	3.66E-45
			$\hat{\alpha}_4$	2.80E-02	2.16E-02	1.29E+00	1.96E-01
			$\hat{\alpha}_3$	-1.65E-02	4.84E-02	-3.42E-01	7.32E-01
		GxEprs_BT*	$\hat{\alpha}_3$	NA	NA	NA	7.22E-01

Table S9: Prediction accuracy and variance explained by GxE component for binary phenotypes across environmental variables

Phenotype	E	AUC of GxEprs_BT	AUC of GxEprs_BT_reduced	P value of ΔAUC	Var(GxE)
OHCAN	BMI	6.1471E-01	6.1420E-01	3.4614E-02	1.0925E-02
	WHR	6.1500E-01	6.1453E-01	4.3510E-02	1.0441E-02
	HD	6.1477E-01	6.1476E-01	7.7762E-01	1.3285E-03
	NS	6.0806E-01	6.0806E-01	9.3884E-01	NA
	BF	6.1637E-01	6.1588E-01	3.5224E-02	1.0684E-02
	PA	6.1222E-01	6.1091E-01	2.6718E-03	1.7165E-02
	WC	6.1770E-01	6.1770E-01	9.3016E-01	NA
	PALC	6.1301E-01	6.1240E-01	2.7995E-02	1.1795E-02
	SMK	6.1445E-01	6.1446E-01	7.4056E-01	NA
HYP	BMI	7.3957E-01	7.3957E-01	5.0009E-01	1.6666E-03
	WHR	7.2615E-01	7.2603E-01	7.6891E-03	6.6120E-03
	HD	7.0472E-01	7.0472E-01	8.3233E-01	3.8993E-04
	NS	7.0830E-01	7.0828E-01	3.5222E-01	2.5804E-03
	BF	7.2808E-01	7.2801E-01	4.4976E-02	4.9619E-03
	PA	7.0711E-01	7.0711E-01	9.6655E-01	NA
	WC	7.3895E-01	7.3895E-01	6.3788E-01	1.1871E-03
	PALC	7.0513E-01	7.0510E-01	2.0840E-01	3.4393E-03
	SMK	7.0687E-01	7.0686E-01	5.6279E-01	1.6276E-03
CAD	BMI	7.3226E-01	7.3201E-01	6.1522E-03	1.0975E-02
	WHR	7.2807E-01	7.2809E-01	9.5061E-01	NA
	HD	7.2101E-01	7.2082E-01	4.4186E-02	9.3369E-03
	NS	7.2355E-01	7.2329E-01	2.5756E-02	1.0825E-02
	BF	7.2806E-01	7.2786E-01	1.4238E-02	9.7089E-03
	PA	7.2225E-01	7.2209E-01	8.4913E-02	8.3152E-03
	WC	7.3060E-01	7.3049E-01	9.7797E-02	7.3043E-03
	PALC	7.2110E-01	7.2019E-01	2.4022E-05	2.0189E-02
	SMK	7.3369E-01	7.3373E-01	4.0434E-01	NA
DIAB	BMI	8.2216E-01	8.2169E-01	5.8316E-05	1.9568E-02
	WHR	7.8925E-01	7.8895E-01	3.2297E-03	1.4483E-02
	HD	7.0190E-01	7.0192E-01	4.9559E-01	NA
	NS	7.0372E-01	7.0375E-01	9.0721E-01	NA
	BF	7.8759E-01	7.8722E-01	1.5801E-03	1.5793E-02
	PA	7.0258E-01	7.0257E-01	5.8397E-01	1.9458E-03
	WC	8.2742E-01	8.2742E-01	4.3775E-01	2.2870E-03
	PALC	7.0343E-01	7.0326E-01	1.1322E-01	8.4654E-03
	SMK	7.1808E-01	7.1805E-01	4.9033E-01	3.7111E-03
HERN	BMI	6.3298E-01	6.3298E-01	8.3559E-01	NA
	WHR	6.3742E-01	6.3733E-01	1.7470E-01	4.5603E-03
	HD	6.3286E-01	6.3286E-01	9.1663E-01	2.3254E-04
	NS	6.3841E-01	6.3770E-01	7.5836E-04	1.2917E-02
	BF	6.3511E-01	6.3505E-01	2.9254E-01	3.6567E-03
	PA	6.3571E-01	6.3564E-01	2.3871E-01	4.2402E-03
	WC	6.3734E-01	6.3730E-01	3.4896E-01	3.2209E-03
	PALC	6.3584E-01	6.3580E-01	4.5881E-01	2.7202E-03
	SMK	6.3038E-01	6.3039E-01	8.1643E-01	NA
DEP	BMI	6.5921E-01	6.5905E-01	1.2664E-01	7.6744E-03
	WHR	6.6283E-01	6.6264E-01	1.3030E-01	8.2486E-03
	HD	6.3374E-01	6.3376E-01	7.6969E-01	NA
	NS	7.7634E-01	7.7626E-01	2.1861E-01	7.5372E-03
	BF	6.5755E-01	6.5738E-01	1.4727E-01	7.7530E-03
	PA	6.2706E-01	6.2707E-01	8.6831E-01	NA
	WC	6.6739E-01	6.6734E-01	3.6914E-01	4.3618E-03
	PALC	6.3663E-01	6.3545E-01	1.6800E-03	1.8867E-02
	SMK	6.5548E-01	6.5327E-01	7.5516E-06	2.7741E-02
CATA	BMI	7.5561E-01	7.5568E-01	8.0390E-01	NA
	WHR	7.5647E-01	7.5625E-01	3.6421E-03	1.0743E-02
	HD	7.5549E-01	7.5549E-01	8.8076E-01	5.6458E-04
	NS	7.5907E-01	7.5908E-01	9.4634E-01	NA
	BF	7.5549E-01	7.5549E-01	7.1238E-01	NA
	PA	7.5903E-01	7.5901E-01	4.5963E-01	3.0389E-03
	WC	7.5658E-01	7.5655E-01	2.1757E-01	3.6895E-03
	PALC	7.5497E-01	7.5483E-01	3.1013E-02	8.6793E-03
	SMK	7.5686E-01	7.5674E-01	5.7771E-02	7.8722E-03
STRO	BMI	7.0575E-01	7.0309E-01	3.0126E-06	4.0442E-02
	WHR	7.0784E-01	7.0372E-01	3.6465E-08	5.0590E-02
	HD	7.0303E-01	7.0193E-01	7.3195E-03	2.5813E-02
	NS	7.1008E-01	7.1036E-01	6.0711E-01	NA
	BF	7.0186E-01	7.0105E-01	1.2178E-02	2.2067E-02
	PA	7.1215E-01	7.1190E-01	2.2583E-01	1.2791E-02
	WC	7.0836E-01	7.0511E-01	2.8436E-07	4.5056E-02
	PALC	6.9952E-01	6.9843E-01	7.5226E-03	2.5449E-02

Continued on next page

Table S9 – *continued from previous page*

Phenotype	E	AUC of GxEprs_BT	AUC of GxEprs_BT_reduced	P value of ΔAUC	Var(GxE)
	SMK	7.0609E-01	7.0624E-01	7.5256E-01	NA
THY	BMI	7.1094E-01	7.1069E-01	4.8358E-02	1.0722E-02
	WHR	7.0144E-01	7.0131E-01	5.9732E-02	7.4979E-03
	HD	6.9951E-01	6.9948E-01	2.5743E-01	3.3222E-03
	NS	6.9792E-01	6.9787E-01	3.7437E-01	4.5685E-03
	BF	7.1059E-01	7.1058E-01	6.5824E-01	2.3055E-03
	PA	7.0209E-01	7.0206E-01	4.4581E-01	3.8429E-03
	WC	7.0943E-01	7.0944E-01	5.1429E-01	NA
	PALC	7.0448E-01	7.0443E-01	4.1690E-01	4.8922E-03
	SMK	7.0062E-01	7.0058E-01	4.3478E-01	4.1871E-03

Phase II: Composite Environmental Variable Analysis

Table S10: Regression summary from the GxE PRS models across each outcome/composite environmental variable pair for quantitative traits

Phenotype	E	Model	Component	Estimate	Standard Error	Test Statistic	P value
BMI	sum_dir	GxEprs_QT	$\hat{\alpha_1}$	-9.87E-02	7.54E-03	-1.31E+01	4.80E-39
			$\hat{\alpha_2}$	2.19E-01	5.40E-03	4.04E+01	0.00E+00
			$\hat{\alpha_3}$	1.00E-02	5.49E-03	1.82E+00	6.84E-02
			$\hat{\alpha_4}$	2.19E-02	7.42E-03	2.95E+00	3.22E-03
		GxEprs_QT*	$\hat{\alpha_4}$	NA	NA	NA	9.00E-03
	PC1	GxEprs_QT	$\hat{\alpha_1}$	-7.83E-02	7.75E-03	-1.01E+01	5.78E-24
			$\hat{\alpha_2}$	2.17E-01	5.39E-03	4.02E+01	0.00E+00
			$\hat{\alpha_3}$	8.46E-03	5.49E-03	1.54E+00	1.23E-01
			$\hat{\alpha_4}$	3.42E-02	7.46E-03	4.59E+00	4.53E-06
		GxEprs_QT*	$\hat{\alpha_4}$	NA	NA	NA	2.00E-05
WHR	sum_dir	GxEprs_QT	$\hat{\alpha_1}$	-1.26E-01	5.54E-03	-2.27E+01	1.93E-113
			$\hat{\alpha_2}$	1.34E-01	4.20E-03	3.20E+01	1.76E-220
			$\hat{\alpha_3}$	6.90E-03	4.19E-03	1.65E+00	1.00E-01
			$\hat{\alpha_4}$	1.06E-02	5.43E-03	1.96E+00	5.03E-02
		GxEprs_QT*	$\hat{\alpha_4}$	NA	NA	NA	5.80E-02
	PC1	GxEprs_QT	$\hat{\alpha_1}$	-1.09E-01	6.66E-03	-1.64E+01	3.53E-60
			$\hat{\alpha_2}$	1.33E-01	4.21E-03	3.17E+01	1.45E-216
			$\hat{\alpha_3}$	8.27E-03	4.22E-03	1.96E+00	5.00E-02
			$\hat{\alpha_4}$	2.17E-02	6.45E-03	3.37E+00	7.51E-04
		GxEprs_QT*	$\hat{\alpha_4}$	NA	NA	NA	1.00E-03
BF	sum_dir	GxEprs_QT	$\hat{\alpha_1}$	-1.07E-01	5.39E-03	-1.98E+01	6.38E-87
			$\hat{\alpha_2}$	1.65E-01	4.17E-03	3.96E+01	0.00E+00
			$\hat{\alpha_3}$	2.36E-03	4.23E-03	5.57E-01	5.77E-01
			$\hat{\alpha_4}$	1.09E-02	5.28E-03	2.06E+00	3.94E-02
		GxEprs_QT*	$\hat{\alpha_4}$	NA	NA	NA	3.90E-02
	PC1	GxEprs_QT	$\hat{\alpha_1}$	-8.34E-02	5.58E-03	-1.50E+01	2.10E-50
			$\hat{\alpha_2}$	1.64E-01	4.20E-03	3.91E+01	0.00E+00
			$\hat{\alpha_3}$	1.16E-03	4.25E-03	2.73E-01	7.85E-01
			$\hat{\alpha_4}$	2.25E-02	5.33E-03	4.23E+00	2.37E-05
		GxEprs_QT*	$\hat{\alpha_4}$	NA	NA	NA	4e-05
WC	sum_dir	GxEprs_QT	$\hat{\alpha_1}$	-1.43E-01	6.14E-03	-2.33E+01	5.32E-119
			$\hat{\alpha_2}$	1.81E-01	4.87E-03	3.73E+01	5.88E-297
			$\hat{\alpha_3}$	4.95E-03	4.93E-03	1.00E+00	3.16E-01
			$\hat{\alpha_4}$	1.70E-02	6.02E-03	2.82E+00	4.85E-03
		GxEprs_QT*	$\hat{\alpha_4}$	NA	NA	NA	9.00E-03
	PC1	GxEprs_QT	$\hat{\alpha_1}$	-1.17E-01	7.07E-03	-1.65E+01	4.56E-61
			$\hat{\alpha_2}$	1.81E-01	4.87E-03	3.71E+01	5.45E-294
			$\hat{\alpha_3}$	6.73E-03	4.95E-03	1.36E+00	1.74E-01
			$\hat{\alpha_4}$	3.05E-02	6.81E-03	4.47E+00	7.74E-06
		GxEprs_QT*	$\hat{\alpha_4}$	NA	NA	NA	5.00E-05

Table S11: Prediction accuracy and variance explained by GxE component for quantitative phenotypes across composite environmental variables

**Phenotype**	**E**	**R^2 of GxEprs_QT**	**R^2 of GxEprs_QT_reduced**	**P value of ΔR^2**	**Var(GxE)**
BMI	PC1	9.5884E-02	9.5200E-02	1.2686E-05	9.4751E-03
	sum_dir	9.8711E-02	9.8430E-02	5.1348E-03	6.0731E-03
WHR	PC1	5.0455E-01	5.0434E-01	1.7604E-02	5.2501E-03
	sum_dir	5.0723E-01	5.0716E-01	1.6931E-01	3.0311E-03
BF	PC1	4.9928E-01	4.9895E-01	2.7549E-03	6.5813E-03
	sum_dir	5.0292E-01	5.0284E-01	1.4616E-01	3.2404E-03
WC	PC1	3.1127E-01	3.1077E-01	2.0287E-04	8.1010E-03
	sum_dir	3.1647E-01	3.1628E-01	1.9797E-02	4.9938E-03

Table S12: Regression summary from the GxE PRS models across each outcome/composite environmental variable pair for binary traits

Phenotype	E	Model	Component	Estimate	Standard Error	Test Statistic	P value
OHCAN	sum_dir	GxEprs_BT	$\hat{\alpha_1}$	-3.45E-01	3.62E-02	-9.52E+00	1.74E-21
			$\hat{\alpha_5}$	-6.44E-02	2.58E-02	-2.50E+00	1.24E-02
			$\hat{\alpha_2}$	1.52E-01	2.71E-02	5.60E+00	2.17E-08
			$\hat{\alpha_4}$	-9.19E-02	2.90E-02	-3.17E+00	1.54E-03
			$\hat{\alpha_3}$	1.09E-02	2.88E-02	3.78E-01	7.06E-01
		GxEprs_BT*	$\hat{\alpha_3}$	NA	NA	NA	7.00E-01
	PC1	GxEprs_BT	$\hat{\alpha_1}$	9.44E-02	3.38E-02	2.80E+00	5.17E-03
			$\hat{\alpha_5}$	-9.67E-02	2.91E-02	-3.32E+00	8.94E-04
			$\hat{\alpha_2}$	1.04E-01	2.53E-02	4.10E+00	4.13E-05
			$\hat{\alpha_4}$	5.28E-02	2.54E-02	2.07E+00	3.81E-02
			$\hat{\alpha_3}$	3.66E-03	2.90E-02	1.26E-01	9.00E-01
		GxEprs_BT*	$\hat{\alpha_3}$	NA	NA	NA	9.85E-01
HYP	sum_dir	GxEprs_BT	$\hat{\alpha_1}$	-5.22E-01	2.65E-02	-1.97E+01	1.10E-86
			$\hat{\alpha_5}$	-4.89E-02	1.75E-02	-2.79E+00	5.26E-03
			$\hat{\alpha_2}$	2.31E-01	1.71E-02	1.35E+01	2.04E-41
			$\hat{\alpha_4}$	-4.83E-02	1.75E-02	-2.76E+00	5.72E-03
			$\hat{\alpha_3}$	2.00E-02	2.37E-02	8.44E-01	3.99E-01
		GxEprs_BT*	$\hat{\alpha_3}$	NA	NA	NA	4.20E-01
	PC1	GxEprs_BT	$\hat{\alpha_1}$	5.86E-01	2.30E-02	2.55E+01	8.99E-144
			$\hat{\alpha_5}$	-2.20E-02	1.71E-02	-1.28E+00	2.00E-01
			$\hat{\alpha_2}$	2.32E-01	1.72E-02	1.35E+01	1.40E-41
			$\hat{\alpha_4}$	6.37E-02	1.75E-02	3.63E+00	2.81E-04
			$\hat{\alpha_3}$	8.54E-03	1.79E-02	4.77E-01	6.33E-01
		GxEprs_BT*	$\hat{\alpha_3}$	NA	NA	NA	6.32E-01
CAD	sum_dir	GxEprs_BT	$\hat{\alpha_1}$	-3.45E-01	3.62E-02	-9.52E+00	1.74E-21
			$\hat{\alpha_5}$	-6.44E-02	2.58E-02	-2.50E+00	1.24E-02
			$\hat{\alpha_2}$	1.52E-01	2.71E-02	5.60E+00	2.17E-08
			$\hat{\alpha_4}$	-9.19E-02	2.90E-02	-3.17E+00	1.54E-03
			$\hat{\alpha_3}$	1.09E-02	2.88E-02	3.78E-01	7.06E-01
		GxEprs_BT*	$\hat{\alpha_3}$	NA	NA	NA	7.00E-01
	PC1	GxEprs_BT	$\hat{\alpha_1}$	3.65E-01	3.79E-02	9.63E+00	5.72E-22
			$\hat{\alpha_5}$	-6.10E-02	2.72E-02	-2.24E+00	2.50E-02
			$\hat{\alpha_2}$	1.53E-01	2.79E-02	5.50E+00	3.81E-08
			$\hat{\alpha_4}$	1.01E-01	3.03E-02	3.32E+00	8.87E-04
			$\hat{\alpha_3}$	1.72E-02	2.71E-02	6.34E-01	5.26E-01
		GxEprs_BT*	$\hat{\alpha_3}$	NA	NA	NA	5.18E-01
DIAB	sum_dir	GxEprs_BT	$\hat{\alpha_1}$	-7.86E-01	4.41E-02	-1.78E+01	5.10E-71
			$\hat{\alpha_5}$	-6.32E-02	2.76E-02	-2.29E+00	2.23E-02
			$\hat{\alpha_2}$	1.94E-01	3.57E-02	5.43E+00	5.63E-08
			$\hat{\alpha_4}$	-9.37E-02	4.11E-02	-2.28E+00	2.27E-02
			$\hat{\alpha_3}$	1.07E-02	2.84E-02	3.77E-01	7.06E-01
		GxEprs_BT*	$\hat{\alpha_3}$	NA	NA	NA	7.00E-01
	PC1	GxEprs_BT	$\hat{\alpha_1}$	1.04E+00	4.69E-02	2.23E+01	8.83E-110
			$\hat{\alpha_5}$	-7.65E-02	2.68E-02	-2.85E+00	4.31E-03
			$\hat{\alpha_2}$	2.35E-01	3.39E-02	6.93E+00	4.21E-12
			$\hat{\alpha_4}$	1.38E-01	4.04E-02	3.41E+00	6.45E-04
			$\hat{\alpha_3}$	2.36E-03	2.90E-02	8.12E-02	9.35E-01
		GxEprs_BT*	$\hat{\alpha_3}$	NA	NA	NA	9.41E-01
HERN	sum_dir	GxEprs_BT	$\hat{\alpha_1}$	-1.43E-01	2.65E-02	-5.39E+00	7.06E-08
			$\hat{\alpha_5}$	-7.12E-02	2.17E-02	-3.29E+00	1.01E-03
			$\hat{\alpha_2}$	8.56E-02	2.04E-02	4.20E+00	2.71E-05
			$\hat{\alpha_4}$	-7.97E-03	2.07E-02	-3.85E-01	7.00E-01
			$\hat{\alpha_3}$	2.32E-02	2.36E-02	9.83E-01	3.26E-01
		GxEprs_BT*	$\hat{\alpha_3}$	NA	NA	NA	3.10E-01
	PC1	GxEprs_BT	$\hat{\alpha_1}$	1.58E-01	2.86E-02	5.53E+00	3.19E-08
			$\hat{\alpha_5}$	-1.27E-01	2.32E-02	-5.49E+00	3.95E-08
			$\hat{\alpha_2}$	8.69E-02	2.05E-02	4.23E+00	2.34E-05
			$\hat{\alpha_4}$	3.34E-02	2.10E-02	1.59E+00	1.11E-01
			$\hat{\alpha_3}$	4.26E-02	2.32E-02	1.84E+00	6.57E-02
		GxEprs_BT*	$\hat{\alpha_3}$	NA	NA	NA	6.50E-02
DEPR	sum_dir	GxEprs_BT	$\hat{\alpha_1}$	-5.21E-01	3.85E-02	-1.35E+01	9.99E-42
			$\hat{\alpha_5}$	-1.38E-02	2.72E-02	-5.05E-01	6.14E-01
			$\hat{\alpha_2}$	5.34E-02	3.33E-02	1.60E+00	1.09E-01
			$\hat{\alpha_4}$	-8.43E-02	3.53E-02	-2.39E+00	1.69E-02
			$\hat{\alpha_3}$	1.53E-02	2.83E-02	5.41E-01	5.88E-01
		GxEprs_BT*	$\hat{\alpha_3}$	NA	NA	NA	5.50E-01
	PC1	GxEprs_BT	$\hat{\alpha_1}$	2.54E-01	3.82E-02	6.65E+00	2.94E-11
			$\hat{\alpha_5}$	3.16E-02	2.73E-02	1.16E+00	2.46E-01
			$\hat{\alpha_2}$	6.24E-02	3.10E-02	2.01E+00	4.41E-02
			$\hat{\alpha_4}$	7.63E-02	3.09E-02	2.47E+00	1.36E-02
			$\hat{\alpha_3}$	-1.17E-02	3.06E-02	-3.82E-01	7.03E-01

Continued on next page

Table S12 – *continued from previous page*

Phenotype	E	Model	Component	Estimate	Standard Error	Test Statistic	P value
		GxEprs_BT*	$\hat{\alpha_3}$	NA	NA	NA	6.78E-01
CATA	sum_dir	GxEprs_BT	$\hat{\alpha_1}$	-1.02E-01	2.63E-02	-3.89E+00	1.02E-04
			$\hat{\alpha_5}$	7.43E-02	2.02E-02	3.67E+00	2.39E-04
			$\hat{\alpha_2}$	1.53E-01	2.23E-02	6.89E+00	5.68E-12
			$\hat{\alpha_4}$	-1.20E-02	2.22E-02	-5.39E-01	5.90E-01
			$\hat{\alpha_3}$	2.62E-03	2.36E-02	1.11E-01	9.12E-01
		GxEprs_BT*	$\hat{\alpha_3}$	NA	NA	NA	8.95E-01
	PC1	GxEprs_BT	$\hat{\alpha_1}$	8.02E-02	2.64E-02	3.04E+00	2.40E-03
			$\hat{\alpha_5}$	7.81E-02	1.93E-02	4.05E+00	5.07E-05
			$\hat{\alpha_2}$	1.50E-01	2.04E-02	7.33E+00	2.24E-13
			$\hat{\alpha_4}$	2.64E-02	2.03E-02	1.30E+00	1.95E-01
			$\hat{\alpha_3}$	-5.32E-03	2.27E-02	-2.34E-01	8.15E-01
		GxEprs_BT*	$\hat{\alpha_3}$	NA	NA	NA	9.82E-01
STRO	sum_dir	GxEprs_BT	$\hat{\alpha_1}$	-7.35E-02	6.27E-02	-1.17E+00	2.41E-01
			$\hat{\alpha_5}$	-1.19E-01	5.25E-02	-2.26E+00	2.35E-02
			$\hat{\alpha_2}$	1.67E-02	4.58E-02	3.64E-01	7.16E-01
			$\hat{\alpha_4}$	-3.22E-02	4.70E-02	-6.83E-01	4.94E-01
			$\hat{\alpha_3}$	-1.28E-01	5.72E-02	-2.25E+00	2.48E-02
		GxEprs_BT*	$\hat{\alpha_3}$	NA	NA	NA	2.30E-02
	PC1	GxEprs_BT	$\hat{\alpha_1}$	7.15E-02	5.90E-02	1.21E+00	2.26E-01
			$\hat{\alpha_5}$	-7.78E-02	4.69E-02	-1.66E+00	9.74E-02
			$\hat{\alpha_2}$	5.71E-02	4.30E-02	1.33E+00	1.83E-01
			$\hat{\alpha_4}$	3.53E-02	4.44E-02	7.94E-01	4.27E-01
			$\hat{\alpha_3}$	-9.35E-02	4.99E-02	-1.87E+00	6.12E-02
		GxEprs_BT*	$\hat{\alpha_3}$	NA	NA	NA	4.90E-02
THY	sum_dir	GxEprs_BT	$\hat{\alpha_1}$	-1.74E-01	3.27E-02	-5.31E+00	1.12E-07
			$\hat{\alpha_5}$	4.00E-03	3.01E-02	1.33E-01	8.94E-01
			$\hat{\alpha_2}$	2.39E-01	3.00E-02	7.99E+00	1.35E-15
			$\hat{\alpha_4}$	-9.54E-03	3.02E-02	-3.16E-01	7.52E-01
			$\hat{\alpha_3}$	-7.90E-03	2.94E-02	-2.69E-01	7.88E-01
		GxEprs_BT*	$\hat{\alpha_3}$	NA	NA	NA	7.90E-01
	PC1	GxEprs_BT	$\hat{\alpha_1}$	1.61E-01	3.40E-02	4.74E+00	2.12E-06
			$\hat{\alpha_5}$	2.33E-02	2.94E-02	7.92E-01	4.29E-01
			$\hat{\alpha_2}$	2.37E-01	2.97E-02	7.98E+00	1.50E-15
			$\hat{\alpha_4}$	-9.75E-03	3.02E-02	-3.23E-01	7.47E-01
			$\hat{\alpha_3}$	-3.06E-02	2.96E-02	-1.03E+00	3.01E-01
		GxEprs_BT*	$\hat{\alpha_3}$	NA	NA	NA	3.19E-01

Table S13: Prediction accuracy and variance explained by GxE component for binary phenotypes across composite environmental variables

Phenotype	E	AUC of GxEprs_BT	AUC of GxEprs_BT_reduced	P value of ΔAUC	Var(GxE)
OHCAN	PC1	6.1770E-01	6.1769E-01	7.6980E-01	1.6871E-03
	sum_dir	6.1776E-01	6.1763E-01	3.9824E-01	5.5423E-03
HYP	PC1	7.4109E-01	7.4107E-01	4.9814E-01	2.4663E-03
	sum_dir	7.4077E-01	7.4075E-01	4.3731E-01	2.8767E-03
CAD	PC1	7.3783E-01	7.3781E-01	5.7402E-01	3.5675E-03
	sum_dir	7.4133E-01	7.4135E-01	9.4154E-01	NA
DIAB	PC1	8.2446E-01	8.2446E-01	9.5198E-01	1.3826E-04
	sum_dir	7.9091E-01	7.9080E-01	1.6776E-01	8.4564E-03
HERN	PC1	6.4147E-01	6.4132E-01	1.9621E-01	5.9219E-03
	sum_dir	6.3882E-01	6.3890E-01	8.9277E-01	NA
DEP	PC1	6.4834E-01	6.4819E-01	3.5626E-01	7.1408E-03
	sum_dir	7.0595E-01	7.0583E-01	3.9059E-01	7.5396E-03
CATA	PC1	7.6258E-01	7.6259E-01	8.6804E-01	NA
	sum_dir	7.6158E-01	7.6158E-01	9.7728E-01	NA
STRO	PC1	7.0988E-01	7.0844E-01	2.4563E-02	3.0258E-02
	sum_dir	7.0531E-01	7.0311E-01	1.1092E-02	3.6732E-02
THY	PC1	7.0970E-01	7.0911E-01	2.5800E-02	1.6609E-02
	sum_dir	7.1102E-01	7.1087E-01	8.7182E-02	8.3892E-03

Phase III: Selected Environmental Variable Analysis

Table S14: Prediction accuracy in terms of R^2 of final model across each outcome for quantitative traits when Step-wise selection method is applied to all the model components

Phenotype	R^2	R^2 P value
BMI	1.25E-01	0.00E+00
WHR	5.18E-01	0.00E+00
BF	5.20E-01	0.00E+00
WC	3.37E-01	0.00E+00

Table S15: AUC of final model across each outcome for binary traits when Step-wise selection method is applied to all the model components

**Phenotype**	**AUC**	**AUC P value**
OHCAN	6.06E-01	4.07E-65
HYP	7.31E-01	0.00E+00
CAD	7.13E-01	0.00E+00
DIAB	7.92E-01	0.00E+00
HERN	6.29E-01	9.22E-177
DEPR	7.78E-01	0.00E+00
CATA	7.41E-01	0.00E+00
STRO	6.81E-01	6.12E-83
THY	6.93E-01	5.65E-205

Notation for Quantitative traits

This notation applies from Notes S1 - S4.

Confounding variables are as follows:
c1: TDI
c2: age
c3 - c12: first 10 genetic principle components
c13: sex
c14: education in years

Ei, prs_addi, prs_gxei and prs_gxei.Ei refer to the ith environmental variable, additive genetic effects of PRS related to ith environmental variable, gxe interaction effects of PRS related to ith environmental variable, and interaction between prs_gxei and Ei respectively, where i = 1, 2, ..., 5. Here 1 = HD, 2 = NS, 3 = PA, 4 = PALC and 5 = SMK.

Step-wise Selection Outputs for each Quantitative Trait

Note S1: Step-wise selection output for BF

```
Call:
lm(formula = y ~ c1 + c2 + c3 + c4 + c5 + c6 + c7 + c8 + c9 +
    c10 + c11 + c12 + c13 + c14 + prs_add1 + E3 + E5 + E1 + prs_gxe4.E4 +
    prs_add3 + E4 + prs_gxe5.E5 + prs_gxe3 + prs_add5 + prs_gxe1 +
    prs_add4 + prs_gxe3.E3, data = data.full)

Residuals:
    Min      1Q  Median      3Q     Max
-3.3263 -0.4405  0.0123  0.4530  3.0986

Coefficients:
              Estimate Std. Error  t value Pr(>|t|)
(Intercept)  7.314e-18  4.183e-03    0.000 1.000000
c1           2.997e-02  4.276e-03    7.008 2.47e-12 ***
c2           1.146e-01  4.315e-03   26.558  < 2e-16 ***
c3           1.919e-03  4.440e-03    0.432 0.665598
c4          -3.250e-03  4.259e-03   -0.763 0.445456
c5          -4.459e-03  4.370e-03   -1.020 0.307556
c6           2.144e-03  6.074e-03    0.353 0.724111
c7           2.347e-02  6.004e-03    3.909 9.29e-05 ***
c8          -3.794e-04  4.315e-03   -0.088 0.929948
c9           8.277e-04  4.391e-03    0.188 0.850490
c10         -2.721e-03  4.610e-03   -0.590 0.555058
c11          4.518e-04  4.422e-03    0.102 0.918625
c12          5.692e-03  4.629e-03    1.230 0.218813
c13         -6.805e-01  4.525e-03 -150.378  < 2e-16 ***
c14         -7.057e-02  4.334e-03  -16.285  < 2e-16 ***
prs_add1     9.674e-02  2.418e-02    4.001 6.32e-05 ***
E3          -9.533e-02  6.511e-03  -14.642  < 2e-16 ***
E5           5.830e-02  4.482e-03   13.007  < 2e-16 ***
E1          -4.789e-02  4.362e-03  -10.979  < 2e-16 ***
prs_gxe4.E4  1.481e-02  4.191e-03    3.534 0.000411 ***
prs_add3     4.730e-02  1.519e-02    3.114 0.001850 **
E4           1.240e-02  4.553e-03    2.723 0.006481 **
prs_gxe5.E5  1.157e-02  4.193e-03    2.759 0.005805 **
prs_gxe3     1.072e-02  4.236e-03    2.531 0.011392 *
prs_add5     3.282e-02  1.537e-02    2.135 0.032739 *
prs_gxe1    -7.341e-03  4.221e-03   -1.739 0.081970 .
prs_add4     2.708e-02  1.643e-02    1.648 0.099348 .
prs_gxe3.E3  9.459e-03  6.481e-03    1.460 0.144420
---
Signif. codes:  0 '***' 0.001 '**' 0.01 '*' 0.05 '.' 0.1 ' ' 1

Residual standard error: 0.6929 on 27416 degrees of freedom
Multiple R-squared:  0.5203, Adjusted R-squared:  0.5199
F-statistic:  1102 on 27 and 27416 DF,  p-value: < 2.2e-16
```

Note S2: Step-wise selection output for BMI

```
Call:
lm(formula = y ~ c1 + c2 + c3 + c4 + c5 + c6 + c7 + c8 + c9 +
    c10 + c11 + c12 + c13 + c14 + prs_add1 + E3 + E5 + E1 + prs_gxe4.E4 +
    prs_gxe5.E5 + prs_add3 + prs_gxe3 + prs_gxe3.E3 + prs_gxe1 +
    prs_add4, data = data.full)

Residuals:
    Min      1Q  Median      3Q     Max
-3.3812 -0.6296 -0.1228  0.4864  8.7408

Coefficients:
              Estimate Std. Error t value Pr(>|t|)
(Intercept)  9.435e-17  5.612e-03   0.000 1.000000
c1           5.108e-02  5.739e-03   8.900  < 2e-16 ***
c2           2.983e-02  5.777e-03   5.163 2.45e-07 ***
c3           2.564e-03  5.956e-03   0.431 0.666822
c4          -7.497e-03  5.714e-03  -1.312 0.189521
c5          -1.084e-02  5.863e-03  -1.848 0.064573 .
c6          -2.526e-03  8.150e-03  -0.310 0.756633
c7           2.466e-02  8.052e-03   3.062 0.002198 **
c8           5.025e-04  5.789e-03   0.087 0.930825
c9           2.189e-03  5.893e-03   0.371 0.710285
c10         -6.215e-03  6.183e-03  -1.005 0.314860
c11         -6.125e-03  5.935e-03  -1.032 0.302086
c12          7.791e-03  6.210e-03   1.255 0.209637
c13          1.025e-01  5.847e-03  17.522  < 2e-16 ***
c14         -8.380e-02  5.809e-03 -14.425  < 2e-16 ***
prs_add1     1.646e-01  2.888e-02   5.699 1.22e-08 ***
E3          -8.610e-02  7.839e-03 -10.984  < 2e-16 ***
E5           6.878e-02  6.393e-03  10.759  < 2e-16 ***
E1          -5.835e-02  5.825e-03 -10.017  < 2e-16 ***
prs_gxe4.E4  2.456e-02  5.632e-03   4.360 1.30e-05 ***
prs_gxe5.E5  2.196e-02  6.136e-03   3.579 0.000346 ***
prs_add3     6.962e-02  2.193e-02   3.175 0.001499 **
prs_gxe3     1.537e-02  5.786e-03   2.657 0.007882 **
prs_gxe3.E3  1.666e-02  7.795e-03   2.137 0.032594 *
prs_gxe1    -1.066e-02  5.659e-03  -1.884 0.059603 .
prs_add4     3.776e-02  2.210e-02   1.709 0.087492 .
---
Signif. codes:  0 '***' 0.001 '**' 0.01 '*' 0.05 '.' 0.1 ' ' 1

Residual standard error: 0.9361 on 27800 degrees of freedom
Multiple R-squared:  0.1245, Adjusted R-squared:  0.1237
F-statistic: 158.1 on 25 and 27800 DF,  p-value: < 2.2e-16
```

Note S3: Step-wise selection output for WC

```
    Call:
lm(formula = y ~ c1 + c2 + c3 + c4 + c5 + c6 + c7 + c8 + c9 +
    c10 + c11 + c12 + c13 + c14 + prs_add1 + E3 + E5 + E1 + prs_gxe4.E4 +
    E4 + prs_add3 + prs_gxe5.E5 + E2 + prs_add5 + prs_gxe3.E3 +
    prs_gxe3 + prs_add4, data = data.full)

Residuals:
    Min      1Q  Median      3Q     Max
-2.8564 -0.5638 -0.0792  0.4725  6.2209

Coefficients:
              Estimate Std. Error t value Pr(>|t|)
(Intercept)  4.139e-16  4.882e-03   0.000  1.00000
c1           4.376e-02  4.998e-03   8.756  < 2e-16 ***
c2           8.673e-02  5.070e-03  17.107  < 2e-16 ***
c3           3.237e-03  5.182e-03   0.625  0.53221
c4          -1.337e-03  4.972e-03  -0.269  0.78794
c5          -6.044e-03  5.101e-03  -1.185  0.23608
c6          -3.355e-03  7.090e-03  -0.473  0.63608
c7          -5.775e-03  6.998e-03  -0.825  0.40925
c8          -6.644e-04  5.036e-03  -0.132  0.89503
c9          -2.766e-03  5.127e-03  -0.540  0.58949
c10          6.203e-04  5.380e-03   0.115  0.90821
c11         -1.404e-02  5.164e-03  -2.720  0.00654 **
c12          2.980e-03  5.403e-03   0.552  0.58129
c13          4.603e-01  5.331e-03  86.348  < 2e-16 ***
c14         -6.098e-02  5.066e-03 -12.037  < 2e-16 ***
prs_add1     1.090e-01  2.520e-02   4.325 1.53e-05 ***
E3          -9.665e-02  6.793e-03 -14.228  < 2e-16 ***
E5           1.015e-01  5.249e-03  19.342  < 2e-16 ***
E1          -5.966e-02  5.095e-03 -11.710  < 2e-16 ***
prs_gxe4.E4  2.479e-02  4.901e-03   5.058 4.27e-07 ***
E4           2.166e-02  5.319e-03   4.071 4.69e-05 ***
prs_add3     4.671e-02  1.649e-02   2.834  0.00460 **
prs_gxe5.E5  1.483e-02  4.893e-03   3.030  0.00245 **
E2           1.329e-02  5.004e-03   2.656  0.00792 **
prs_add5     3.942e-02  1.595e-02   2.471  0.01347 *
prs_gxe3.E3  1.446e-02  6.752e-03   2.142  0.03219 *
prs_gxe3     9.452e-03  4.937e-03   1.914  0.05559 .
prs_add4     2.705e-02  1.705e-02   1.586  0.11269
---
Signif. codes:  0 '***' 0.001 '**' 0.01 '*' 0.05 '.' 0.1 ' ' 1

Residual standard error: 0.8145 on 27799 degrees of freedom
Multiple R-squared:  0.3373, Adjusted R-squared:  0.3367
F-statistic: 524.1 on 27 and 27799 DF,  p-value: < 2.2e-16
```

Note S4: Step-wise selection output for WHR

```
    Call:
lm(formula = y ~ c1 + c2 + c3 + c4 + c5 + c6 + c7 + c8 + c9 +
    c10 + c11 + c12 + c13 + c14 + prs_add1 + E5 + E3 + E1 + E4 +
    E2 + prs_add5 + prs_add4 + prs_gxe4.E4 + prs_gxe5.E5 + prs_add2 +
    prs_gxe1.E1, data = data.full)

Residuals:
    Min      1Q  Median      3Q     Max
-3.0614 -0.4722 -0.0350  0.4333  6.5672

Coefficients:
              Estimate Std. Error t value Pr(>|t|)
(Intercept) -3.425e-17  4.165e-03   0.000 1.000000
c1           3.546e-02  4.264e-03   8.315  < 2e-16 ***
c2           1.186e-01  4.325e-03  27.416  < 2e-16 ***
c3          -4.109e-03  4.421e-03  -0.929 0.352652
c4           6.555e-03  4.242e-03   1.545 0.122268
c5          -2.599e-03  4.352e-03  -0.597 0.550325
c6           8.605e-04  6.050e-03   0.142 0.886897
c7           4.717e-03  5.969e-03   0.790 0.429427
c8          -5.328e-03  4.296e-03  -1.240 0.214928
c9          -5.461e-03  4.373e-03  -1.249 0.211791
c10          4.374e-03  4.589e-03   0.953 0.340603
c11         -1.476e-02  4.405e-03  -3.350 0.000809 ***
c12          6.446e-03  4.609e-03   1.399 0.161936
c13          6.373e-01  4.548e-03 140.131  < 2e-16 ***
c14         -5.767e-02  4.323e-03 -13.341  < 2e-16 ***
prs_add1     7.075e-02  1.985e-02   3.565 0.000365 ***
E5           1.047e-01  5.331e-03  19.648  < 2e-16 ***
E3          -6.058e-02  4.214e-03 -14.375  < 2e-16 ***
E1          -4.693e-02  4.572e-03 -10.263  < 2e-16 ***
E4           2.666e-02  5.401e-03   4.937 7.99e-07 ***
E2           2.150e-02  4.270e-03   5.035 4.81e-07 ***
prs_add5     3.513e-02  1.215e-02   2.892 0.003836 **
prs_add4     3.281e-02  1.383e-02   2.372 0.017708 *
prs_gxe4.E4  1.182e-02  5.104e-03   2.315 0.020633 *
prs_gxe5.E5  1.048e-02  5.075e-03   2.065 0.038908 *
prs_add2     2.365e-02  1.362e-02   1.736 0.082619 .
prs_gxe1.E1  7.299e-03  4.406e-03   1.657 0.097598 .
---
Signif. codes:  0 '***' 0.001 '**' 0.01 '*' 0.05 '.' 0.1 ' ' 1

Residual standard error: 0.6948 on 27796 degrees of freedom
Multiple R-squared:  0.5177, Adjusted R-squared:  0.5173
F-statistic:  1148 on 26 and 27796 DF,  p-value: < 2.2e-16
```

Notation for Binary traits

This notation applies from Notes S5 - S13.

Confounding variables are as follows:
c1: TDI
c2: age
c3 - c12: first 10 genetic principle components
c13: sex
c14: education in years

Ei, Ei_sq prs_addi, prs_gxei and prs_gxei.Ei refer to the ith environmental variable, square of ith environmental variable, additive genetic effects of PRS related to ith environmental variable, gxe interaction effects of PRS related to ith environmental variable, and interaction between prs_gxei and Ei respectively, where i = 1, 2, ..., 9. Here 1 = BMI, 2 = WHR, 3 = HD, 4 = NS, 5 = BF, 6 = PA, 7 = WC, 8 = PALC and 9 = SMK.

Step-wise Selection Outputs for each Binary Trait

Note S5: Step-wise selection output for CAD

```
Call:
glm(formula = y ~ c1 + c2 + c3 + c4 + c5 + c6 + c7 + c8 + c9 +
    c10 + c11 + c12 + c13 + c14 + E1 + E9 + prs_add6 + E2 + E9_sq +
    E4 + E7 + E2_sq + prs_gxe8.E8 + prs_gxe1.E1 + prs_gxe6 +
    E1_sq + prs_add2 + E8_sq, family = binomial(link = "logit"),
    data = data.full)

Deviance Residuals:
    Min       1Q   Median       3Q      Max
-1.1072  -0.3986  -0.2820  -0.1930   3.3174

Coefficients:
             Estimate Std. Error z value Pr(>|z|)
(Intercept) -3.083104   0.034318 -89.840  < 2e-16 ***
c1           0.045155   0.025789   1.751 0.079963 .
c2           0.624401   0.031680  19.710  < 2e-16 ***
c3          -0.006508   0.027485  -0.237 0.812824
c4          -0.015051   0.026457  -0.569 0.569434
c5           0.029643   0.027173   1.091 0.275307
c6           0.002672   0.037786   0.071 0.943617
c7           0.033657   0.037072   0.908 0.363947
c8          -0.019583   0.026669  -0.734 0.462762
c9           0.029846   0.027470   1.086 0.277260
c10         -0.013099   0.028675  -0.457 0.647801
c11          0.068862   0.028050   2.455 0.014089 *
c12          0.039098   0.028417   1.376 0.168861
c13          0.339778   0.039659   8.567  < 2e-16 ***
c14         -0.076161   0.026087  -2.919 0.003507 **
E1           0.404380   0.066531   6.078 1.22e-09 ***
E9           0.256051   0.034811   7.355 1.90e-13 ***
prs_add6     0.140276   0.046243   3.033 0.002417 **
E2           0.333611   0.068995   4.835 1.33e-06 ***
E9_sq       -0.089584   0.029146  -3.074 0.002114 **
E4           0.091447   0.026263   3.482 0.000498 ***
E7          -0.258864   0.091312  -2.835 0.004583 **
E2_sq       -0.056316   0.028547  -1.973 0.048522 *
prs_gxe8.E8  0.046235   0.025885   1.786 0.074073 .
prs_gxe1.E1  0.065954   0.031615   2.086 0.036963 *
prs_gxe6     0.050216   0.026114   1.923 0.054482 .
E1_sq       -0.055139   0.030621  -1.801 0.071749 .
prs_add2     0.080936   0.046375   1.745 0.080942 .
E8_sq       -0.040297   0.029801  -1.352 0.176304
---
Signif. codes:  0 '***' 0.001 '**' 0.01 '*' 0.05 '.' 0.1 ' ' 1
```

Note S6: Step-wise selection output for CATA

```
    Call:
glm(formula = y ~ c1 + c2 + c3 + c4 + c5 + c6 + c7 + c8 + c9 +
    c10 + c11 + c12 + c13 + c14 + prs_add5 + E9 + E7_sq + E4 +
    E9_sq + E8_sq + E7 + prs_gxe5.E5 + prs_gxe9.E9 + prs_gxe3 +
    prs_gxe5 + prs_gxe1 + prs_gxe7 + prs_add3 + prs_add2 + prs_add8,
    family = binomial(link = "logit"), data = data.full)

Deviance Residuals:
    Min       1Q   Median       3Q      Max
-1.2537  -0.4973  -0.3236  -0.1836   3.2870

Coefficients:
             Estimate Std. Error z value Pr(>|z|)
(Intercept) -2.754669   0.030717 -89.678  < 2e-16 ***
c1           0.056470   0.022354   2.526 0.011533 *
c2           1.091310   0.030793  35.441  < 2e-16 ***
c3          -0.004959   0.023353  -0.212 0.831845
c4          -0.016461   0.022366  -0.736 0.461727
c5          -0.014728   0.023106  -0.637 0.523855
c6           0.025167   0.032121   0.783 0.433341
c7          -0.031628   0.031783  -0.995 0.319669
c8           0.010725   0.022667   0.473 0.636110
c9           0.003063   0.023184   0.132 0.894878
c10          0.034818   0.024273   1.434 0.151445
c11          0.074245   0.024219   3.066 0.002173 **
c12         -0.034436   0.024130  -1.427 0.153543
c13         -0.196602   0.026798  -7.337 2.19e-13 ***
c14          0.013746   0.022069   0.623 0.533388
prs_add5     0.211550   0.158609   1.334 0.182273
E9           0.151510   0.032312   4.689 2.75e-06 ***
E7_sq        0.070486   0.020960   3.363 0.000771 ***
E4           0.067903   0.022534   3.013 0.002583 **
E9_sq       -0.075404   0.029797  -2.531 0.011386 *
E8_sq        0.043824   0.016964   2.583 0.009784 **
E7           0.068144   0.026992   2.525 0.011584 *
prs_gxe5.E5 -0.047101   0.023130  -2.036 0.041715 *
prs_gxe9.E9  0.030391   0.018299   1.661 0.096744 .
prs_gxe3     0.045339   0.023213   1.953 0.050802 .
prs_gxe5    -0.072977   0.040764  -1.790 0.073415 .
prs_gxe1     0.137017   0.050595   2.708 0.006767 **
prs_gxe7    -0.115513   0.046701  -2.473 0.013381 *
prs_add3     0.233829   0.125795   1.859 0.063054 .
prs_add2    -0.323322   0.189247  -1.708 0.087549 .
prs_add8     0.087003   0.060693   1.433 0.151721
---
Signif. codes:  0 '***' 0.001 '**' 0.01 '*' 0.05 '.' 0.1 ' ' 1
```

Note S7: Step-wise selection output for DEPR

```
    Call:
glm(formula = y ~ c1 + c2 + c3 + c4 + c5 + c6 + c7 + c8 + c9 +
    c10 + c11 + c12 + c13 + c14 + E4 + E2 + E9 + E1_sq + prs_add5 +
    prs_gxe8.E8 + E8_sq + prs_gxe8 + prs_gxe9.E9 + E9_sq + prs_gxe4.E4 +
    E2_sq + E1 + E5 + E8, family = binomial(link = "logit"),
    data = data.full)

Deviance Residuals:
    Min       1Q   Median       3Q      Max
-1.9325  -0.3019  -0.2070  -0.1489   3.2734

Coefficients:
              Estimate Std. Error z value Pr(>|z|)
(Intercept) -3.626e+00  4.259e-02 -85.130  < 2e-16 ***
c1           1.334e-01  2.978e-02   4.479 7.48e-06 ***
c2           2.550e-02  3.410e-02   0.748  0.45463
c3           4.429e-02  3.335e-02   1.328  0.18412
c4           3.926e-02  3.232e-02   1.215  0.22443
c5           4.893e-02  3.309e-02   1.479  0.13921
c6           4.463e-02  4.582e-02   0.974  0.33006
c7          -1.444e-01  4.561e-02  -3.167  0.00154 **
c8           1.760e-02  3.254e-02   0.541  0.58867
c9          -1.413e-02  3.312e-02  -0.427  0.66956
c10          8.848e-05  3.478e-02   0.003  0.99797
c11          3.258e-02  3.543e-02   0.920  0.35781
c12          1.734e-02  3.489e-02   0.497  0.61916
c13         -4.857e-01  7.920e-02  -6.132 8.65e-10 ***
c14         -5.822e-02  3.248e-02  -1.792  0.07307 .
E4           7.744e-01  3.720e-02  20.816  < 2e-16 ***
E2           3.270e-01  4.982e-02   6.563 5.28e-11 ***
E9           3.037e-01  6.182e-02   4.912 9.03e-07 ***
E1_sq        2.915e-02  2.868e-02   1.016  0.30942
prs_add5     7.870e-02  3.179e-02   2.476  0.01330 *
prs_gxe8.E8  4.479e-02  6.772e-02   0.661  0.50832
E8_sq        9.254e-02  2.872e-02   3.222  0.00127 **
prs_gxe8     6.107e-02  3.175e-02   1.924  0.05437 .
prs_gxe9.E9  9.637e-02  4.744e-02   2.032  0.04220 *
E9_sq       -6.678e-02  3.872e-02  -1.725  0.08458 .
prs_gxe4.E4  5.636e-02  3.232e-02   1.744  0.08116 .
E2_sq       -4.632e-02  2.996e-02  -1.546  0.12216
E1           1.817e-01  6.782e-02   2.680  0.00737 **
E5          -1.837e-01  8.303e-02  -2.213  0.02692 *
E8          -1.041e-01  7.261e-02  -1.433  0.15183
---
Signif. codes:  0 '***' 0.001 '**' 0.01 '*' 0.05 '.' 0.1 ' ' 1
```

Note S8: Step-wise selection output for DIAB

```
    Call:
glm(formula = y ~ c1 + c2 + c3 + c4 + c5 + c6 + c7 + c8 + c9 +
    c10 + c11 + c12 + c13 + c14 + E7 + prs_add5 + E9 + E2 + E1 +
    E8 + E2_sq + E5_sq + E5 + prs_gxe8.E8 + prs_gxe5 + E4 + prs_add1 +
    prs_gxe1.E1 + prs_gxe1, family = binomial(link = "logit"),
    data = data.full)

Deviance Residuals:
    Min       1Q   Median       3Q      Max
-1.8454  -0.3307  -0.2186  -0.1393   3.5332

Coefficients:
             Estimate Std. Error z value Pr(>|z|)
(Intercept) -3.578377   0.044865 -79.759  < 2e-16 ***
c1           0.118163   0.028257   4.182 2.89e-05 ***
c2           0.432119   0.035178  12.284  < 2e-16 ***
c3          -0.013707   0.031336  -0.437 0.661799
c4           0.042758   0.030187   1.416 0.156651
c5           0.066489   0.030973   2.147 0.031818 *
c6          -0.001755   0.042994  -0.041 0.967441
c7          -0.007778   0.042523  -0.183 0.854863
c8          -0.041803   0.030352  -1.377 0.168421
c9           0.042108   0.031353   1.343 0.179259
c10         -0.047793   0.032615  -1.465 0.142823
c11          0.058790   0.031218   1.883 0.059670 .
c12          0.077993   0.032143   2.426 0.015249 *
c13         -0.282950   0.080219  -3.527 0.000420 ***
c14         -0.065873   0.029690  -2.219 0.026509 *
E7           0.008746   0.097644   0.090 0.928628
prs_add5    -0.088030   0.133931  -0.657 0.511003
E9           0.148270   0.022377   6.626 3.45e-11 ***
E2           0.695741   0.077431   8.985  < 2e-16 ***
E1           0.673290   0.070530   9.546  < 2e-16 ***
E8          -0.197668   0.049734  -3.974 7.05e-05 ***
E2_sq       -0.100152   0.030311  -3.304 0.000953 ***
E5_sq       -0.113325   0.034434  -3.291 0.000998 ***
E5          -0.252905   0.082098  -3.081 0.002066 **
prs_gxe8.E8 -0.114553   0.044405  -2.580 0.009889 **
prs_gxe5    -0.006839   0.050926  -0.134 0.893167
E4           0.057749   0.029613   1.950 0.051159 .
prs_add1     0.405588   0.150044   2.703 0.006869 **
prs_gxe1.E1  0.047196   0.026103   1.808 0.070598 .
prs_gxe1     0.141420   0.085569   1.653 0.098393 .
---
Signif. codes:  0 '***' 0.001 '**' 0.01 '*' 0.05 '.' 0.1 ' ' 1
```

Note S9: Step-wise selection output for HERN

```
    Call:
glm(formula = y ~ c1 + c2 + c3 + c4 + c5 + c6 + c7 + c8 + c9 +
    c10 + c11 + c12 + c13 + c14 + E2 + prs_add5 + E4 + E7_sq +
    E5_sq + prs_gxe7.E7 + prs_gxe4.E4 + E8 + E8_sq + prs_gxe9.E9 +
    E5 + prs_gxe6.E6, family = binomial(link = "logit"), data = data.full)

Deviance Residuals:
    Min       1Q   Median       3Q      Max
-1.1489  -0.5306  -0.4318  -0.3331   2.9364

Coefficients:
              Estimate Std. Error  z value Pr(>|z|)
(Intercept) -2.2257665  0.0222458 -100.053  < 2e-16 ***
c1           0.0467194  0.0201343    2.320 0.020320 *
c2           0.3576427  0.0223696   15.988  < 2e-16 ***
c3           0.0004014  0.0214525    0.019 0.985070
c4           0.0176513  0.0206192    0.856 0.391965
c5          -0.0040059  0.0211850   -0.189 0.850020
c6           0.0283615  0.0295499    0.960 0.337163
c7           0.0104462  0.0289786    0.360 0.718488
c8           0.0014861  0.0208681    0.071 0.943227
c9           0.0290213  0.0214408    1.354 0.175878
c10         -0.0065313  0.0223417   -0.292 0.770028
c11          0.0689494  0.0221889    3.107 0.001887 **
c12          0.0147011  0.0222814    0.660 0.509387
c13          0.2102829  0.0437716    4.804 1.55e-06 ***
c14         -0.0676549  0.0205283   -3.296 0.000982 ***
E2           0.1980794  0.0329830    6.006 1.91e-09 ***
prs_add5     0.1308332  0.0202781    6.452 1.10e-10 ***
E4           0.0939473  0.0223186    4.209 2.56e-05 ***
E7_sq       -0.1119662  0.0223804   -5.003 5.65e-07 ***
E5_sq        0.0883405  0.0198690    4.446 8.74e-06 ***
prs_gxe7.E7  0.0523456  0.0217889    2.402 0.016288 *
prs_gxe4.E4 -0.0463022  0.0219029   -2.114 0.034517 *
E8          -0.0776637  0.0264519   -2.936 0.003324 **
E8_sq        0.0508925  0.0204291    2.491 0.012732 *
prs_gxe9.E9 -0.0314224  0.0174642   -1.799 0.071980 .
E5           0.0619062  0.0334194    1.852 0.063968 .
prs_gxe6.E6 -0.0354723  0.0189930   -1.868 0.061810 .
---
Signif. codes:  0 '***' 0.001 '**' 0.01 '*' 0.05 '.' 0.1 ' ' 1
```

Note S10: Step-wise selection output for HYP

```
    Call:
glm(formula = y ~ c1 + c2 + c3 + c4 + c5 + c6 + c7 + c8 + c9 +
    c10 + c11 + c12 + c13 + c14 + E1 + prs_add1 + E2 + E8 + E4 +
    E9 + prs_gxe2.E2 + prs_gxe7 + E7_sq + E8_sq + E5 + E5_sq +
    prs_add5 + prs_gxe3.E3, family = binomial(link = "logit"),
    data = data.full)

Deviance Residuals:
    Min       1Q   Median       3Q      Max
-2.0192  -0.7108  -0.4794  -0.2522   3.0273

Coefficients:
             Estimate Std. Error z value Pr(>|z|)
(Intercept) -1.564521   0.018826 -83.102  < 2e-16 ***
c1           0.049864   0.016667   2.992  0.00277 **
c2           0.704638   0.019704  35.762  < 2e-16 ***
c3          -0.006564   0.017371  -0.378  0.70552
c4           0.008361   0.016735   0.500  0.61734
c5          -0.002445   0.017177  -0.142  0.88681
c6          -0.046482   0.023907  -1.944  0.05187 .
c7          -0.036836   0.023767  -1.550  0.12118
c8           0.004631   0.016879   0.274  0.78380
c9           0.009628   0.017271   0.557  0.57720
c10         -0.013826   0.018043  -0.766  0.44350
c11          0.013375   0.017219   0.777  0.43731
c12          0.029233   0.017996   1.624  0.10428
c13         -0.097051   0.042734  -2.271  0.02314 *
c14         -0.103799   0.016579  -6.261 3.83e-10 ***
E1           0.479966   0.034420  13.945  < 2e-16 ***
prs_add1     0.393382   0.085201   4.617 3.89e-06 ***
E2           0.228769   0.027693   8.261  < 2e-16 ***
E8           0.130612   0.021828   5.984 2.18e-09 ***
E4           0.098499   0.016715   5.893 3.79e-09 ***
E9           0.075668   0.015443   4.900 9.59e-07 ***
prs_gxe2.E2  0.043331   0.018056   2.400  0.01640 *
prs_gxe7     0.049217   0.019219   2.561  0.01044 *
E7_sq       -0.043504   0.017483  -2.488  0.01283 *
E8_sq       -0.030610   0.018629  -1.643  0.10034
E5          -0.093746   0.043962  -2.132  0.03297 *
E5_sq       -0.032939   0.017991  -1.831  0.06712 .
prs_add5    -0.129125   0.083896  -1.539  0.12378
prs_gxe3.E3 -0.024680   0.016394  -1.505  0.13221
---
Signif. codes:  0 '***' 0.001 '**' 0.01 '*' 0.05 '.' 0.1 ' ' 1
```

Note S11: Step-wise selection output for OHCAN

```
Call:
glm(formula = y ~ c1 + c2 + c3 + c4 + c5 + c6 + c7 + c8 + c9 +
    c10 + c11 + c12 + c13 + c14 + prs_add1 + E7_sq + E5 + E5_sq +
    E2_sq + prs_gxe6.E6 + prs_gxe2 + prs_add5 + prs_gxe5.E5,
    family = binomial(link = "logit"), data = data.full)

Deviance Residuals:
    Min       1Q   Median       3Q      Max
-0.6898  -0.3731  -0.3152  -0.2567   2.8981

Coefficients:
             Estimate Std. Error z value Pr(>|z|)
(Intercept) -2.956128   0.029565 -99.989  < 2e-16 ***
c1          -0.002932   0.027896  -0.105  0.91628
c2           0.395410   0.030388  13.012  < 2e-16 ***
c3           0.028131   0.028720   0.979  0.32733
c4          -0.028808   0.027641  -1.042  0.29731
c5           0.002345   0.028401   0.083  0.93418
c6          -0.039423   0.039198  -1.006  0.31454
c7           0.023843   0.039114   0.610  0.54215
c8           0.065479   0.027977   2.340  0.01926 *
c9          -0.018941   0.028471  -0.665  0.50587
c10         -0.044466   0.029947  -1.485  0.13759
c11          0.042785   0.028822   1.484  0.13769
c12          0.079124   0.029820   2.653  0.00797 **
c13          0.030252   0.038822   0.779  0.43584
c14          0.056021   0.027604   2.029  0.04241 *
prs_add1     0.453441   0.218207   2.078  0.03771 *
E7_sq       -0.164059   0.040891  -4.012 6.02e-05 ***
E5           0.116203   0.039117   2.971  0.00297 **
E5_sq        0.069954   0.027561   2.538  0.01114 *
E2_sq        0.091260   0.035351   2.582  0.00984 **
prs_gxe6.E6 -0.052439   0.025936  -2.022  0.04319 *
prs_gxe2     0.064401   0.028182   2.285  0.02230 *
prs_add5    -0.330980   0.218149  -1.517  0.12921
prs_gxe5.E5  0.040655   0.027434   1.482  0.13837
---
Signif. codes:  0 '***' 0.001 '**' 0.01 '*' 0.05 '.' 0.1 ' ' 1
```

Note S12: Step-wise selection output for STRO

```
    Call:
glm(formula = y ~ c1 + c2 + c3 + c4 + c5 + c6 + c7 + c8 + c9 +
    c10 + c11 + c12 + c13 + c14 + E9 + E9_sq + prs_gxe2.E2 +
    E7_sq + E4_sq + prs_add7 + E5_sq, family = binomial(link = "logit"),
    data = data.full)

Deviance Residuals:
    Min       1Q   Median       3Q      Max
-0.6373  -0.2263  -0.1691  -0.1198   3.4812

Coefficients:
            Estimate Std. Error z value Pr(>|z|)
(Intercept) -4.25494    0.05779 -73.621  < 2e-16 ***
c1          -0.01120    0.04568  -0.245 0.806341
c2           0.68894    0.05650  12.193  < 2e-16 ***
c3           0.02191    0.04748   0.462 0.644393
c4           0.02841    0.04546   0.625 0.531974
c5           0.01001    0.04708   0.213 0.831642
c6          -0.05313    0.06489  -0.819 0.412930
c7           0.05309    0.06423   0.827 0.408484
c8           0.00111    0.04602   0.024 0.980762
c9           0.05024    0.04739   1.060 0.289062
c10          0.02599    0.04938   0.526 0.598624
c11          0.05580    0.04827   1.156 0.247748
c12          0.04115    0.04907   0.839 0.401646
c13          0.08542    0.04865   1.756 0.079149 .
c14         -0.06117    0.04498  -1.360 0.173890
E9           0.32725    0.06673   4.904 9.38e-07 ***
E9_sq       -0.20584    0.07234  -2.845 0.004435 **
prs_gxe2.E2 -0.17343    0.04712  -3.681 0.000232 ***
E7_sq       -0.10404    0.05096  -2.041 0.041204 *
E4_sq        0.08754    0.04250   2.060 0.039432 *
prs_add7     0.06902    0.04452   1.550 0.121099
E5_sq        0.06527    0.04466   1.462 0.143873
---
Signif. codes:  0 '***' 0.001 '**' 0.01 '*' 0.05 '.' 0.1 ' ' 1
```

Note S13: Step-wise selection output for THY

```
Call:
glm(formula = y ~ c1 + c2 + c3 + c4 + c5 + c6 + c7 + c8 + c9 +
    c10 + c11 + c12 + c13 + c14 + prs_add3 + E7 + E8 + E4 + E8_sq +
    E9 + E2 + E9_sq + E3 + E7_sq + prs_add9, family = binomial(link = "logit"),
    data = data.full)

Deviance Residuals:
    Min       1Q   Median       3Q      Max
-1.0133  -0.3396  -0.2481  -0.1797   3.3159

Coefficients:
            Estimate Std. Error z value Pr(>|z|)
(Intercept) -3.37716    0.03798 -88.930  < 2e-16 ***
c1           0.02967    0.03045   0.974 0.329959
c2           0.34211    0.03401  10.061  < 2e-16 ***
c3           0.05129    0.03161   1.623 0.104636
c4          -0.02025    0.03044  -0.665 0.506014
c5           0.06516    0.03145   2.072 0.038285 *
c6          -0.03796    0.04315  -0.880 0.378933
c7           0.01272    0.04282   0.297 0.766412
c8           0.02486    0.03087   0.805 0.420638
c9           0.02134    0.03117   0.685 0.493640
c10          0.01591    0.03293   0.483 0.628953
c11          0.04733    0.03228   1.466 0.142542
c12          0.05027    0.03286   1.530 0.126037
c13         -0.56287    0.04561 -12.342  < 2e-16 ***
c14         -0.09062    0.03084  -2.938 0.003300 **
prs_add3     0.22615    0.07324   3.088 0.002017 **
E7           0.31319    0.05174   6.053 1.42e-09 ***
E8          -0.23936    0.04609  -5.193 2.07e-07 ***
E4           0.10523    0.02995   3.514 0.000442 ***
E8_sq        0.13157    0.02822   4.663 3.12e-06 ***
E9           0.17056    0.04858   3.511 0.000447 ***
E2          -0.18337    0.06164  -2.975 0.002932 **
E9_sq       -0.07933    0.04937  -1.607 0.108139
E3           0.05633    0.03182   1.770 0.076667 .
E7_sq       -0.04503    0.03070  -1.467 0.142442
prs_add9     0.10499    0.07314   1.436 0.151136
---
Signif. codes:  0 '***' 0.001 '**' 0.01 '*' 0.05 '.' 0.1 ' ' 1
```

Table S16: GWEIS model specifications and plots

Phenotype	E	Effects	QQ Plot	Manhattan Plot	Genomic Inflation Factor (λ)	Scaled Genomic Inflation Factor (λ1000)	Theoretical Genomic Inflation Factor (lapprox)	GxE Significance Identified
BMI	PALC	Additive genetic effects	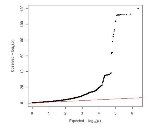	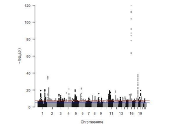	1.6303E+00	1.0027E+00	1.0567E+00	Pase I, Phase III
		GxE interaction effects	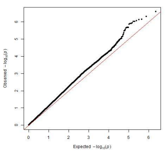	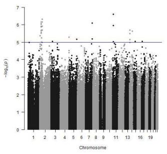	1.1487E+00	1.0006E+00		
BMI	SMK	Additive genetic effects	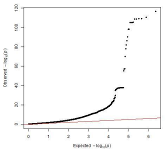	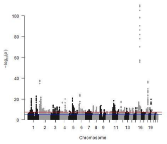	1.6056E+00	1.0026E+00	1.0929E+00	Pase I, Phase III
		GxE interaction effects	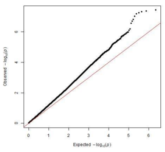	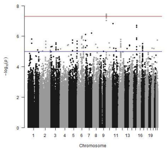	1.2380E+00	1.0010E+00		
BMI	PA	Additive genetic effects	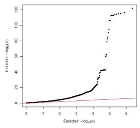	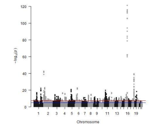	1.6285E+00	1.0027E+00	9.7594E-01	Phase III
		GxE interaction effects	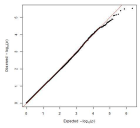	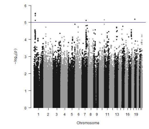	9.6596E-01	9.9985E-01		
WHR	PALC	Additive genetic effects	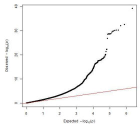	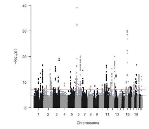	1.4455E+00	1.0019E+00	1.0276E+00	Pase I, Phase III
		GxE interaction effects	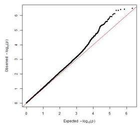	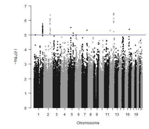	1.0614E+00	1.0003E+00		
WHR	SMK	Additive genetic effects	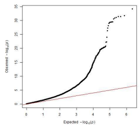	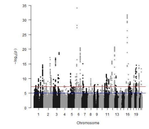	1.4221E+00	1.0018E+00	1.0597E+00	Phase III
		GxE interaction effects	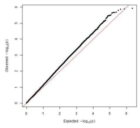	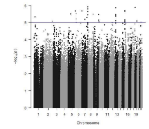	1.1316E+00	1.0006E+00		

BF	PALC	Additive genetic effects	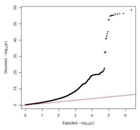	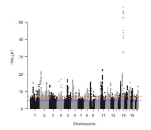	1.6122E+00	1.0027E+00	1.0142E+00	Pase I, Phase III
		GxE interaction effects	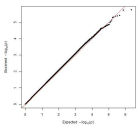	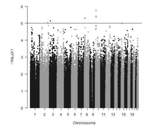	1.0451E+00	1.0002E+00		
BF	SMK	Additive genetic effects	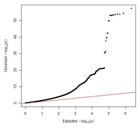	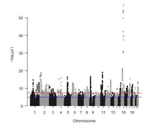	1.5868E+00	1.0026E+00	1.0128E+00	Pase I, Phase III
		GxE interaction effects	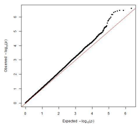	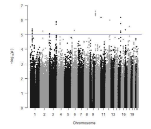	1.0625E+00	1.0003E+00		
WC	PALC	Additive genetic effects	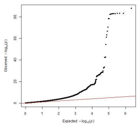	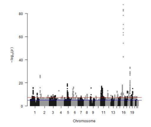	1.5413E+00	1.0024E+00	1.0646E+00	Pase I, Phase III
		GxE interaction effects	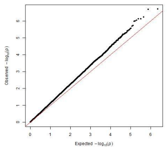	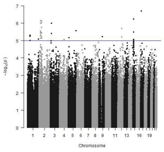	1.1557E+00	1.0007E+00		
WC	SMK	Additive genetic effects	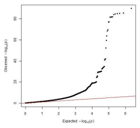	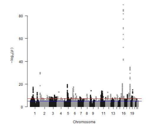	1.5269E+00	1.0023E+00	1.1064E+00	Phase III
		GxE interaction effects	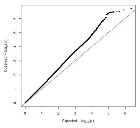	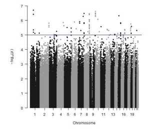	1.2534E+00	1.0011E+00		
WC	PA	Additive genetic effects	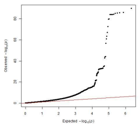	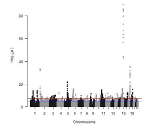	1.5592E+00	1.0024E+00	1.0037E+00	Phase III
		GxE interaction effects	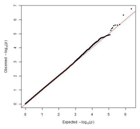	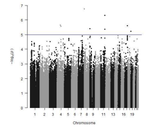	1.0216E+00	1.0001E+00		
CAD	BMI	Additive genetic effects	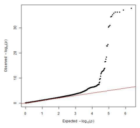	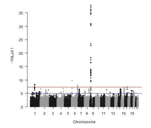	1.0930E+00	1.0032E+00	1.0064E+00	Phase III
		GxE interaction effects	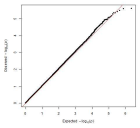	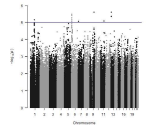	1.0198E+00	1.0007E+00		

CATA	BF	Additive genetic effects	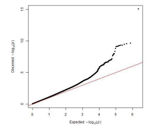	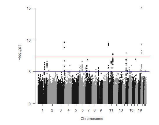	1.0871E+00	1.0021E+00	1.0024E+00	Phase III
		GxE interaction effects	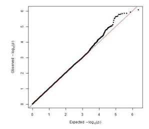	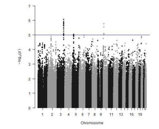	1.0079E+00	1.0002E+00		
DEP	SMK	Additive genetic effects	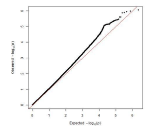	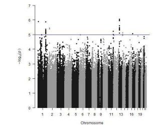	1.0374E+00	1.0017E+00	1.0025E+00	Phase III
		GxE interaction effects	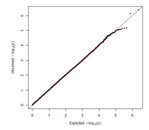	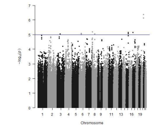	1.0073E+00	1.0003E+00		
DIAB	PALC	Additive genetic effects	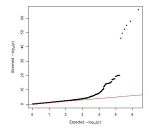	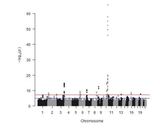	1.1231E+00	1.0049E+00	1.2260E+00	Phase III
		GxE interaction effects	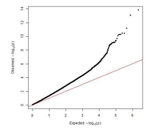	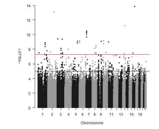	1.4454E+00	1.0179E+00		
HERN	NS	Additive genetic effects	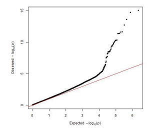	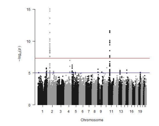	1.0871E+00	1.0020E+00	1.0021E+00	Phase III
		GxE interaction effects	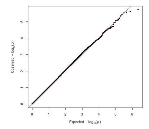	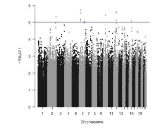	1.0141E+00	1.0003E+00		
HYP	WHR	Additive genetic effects	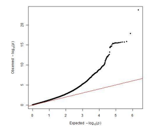	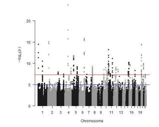	1.2234E+00	1.0029E+00	1.0207E+00	Phase III
		GxE interaction effects	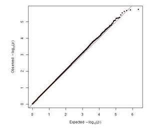	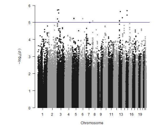	1.0456E+00	1.0006E+00		

OHCAN	PA	Additive genetic effects	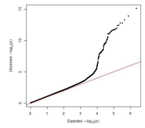	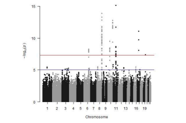	1.0413E+00	1.0018E+00	9.9859E-01	Phase III
		GxE interaction effects	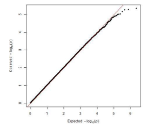	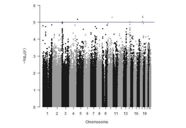	1.0061E+00	1.0003E+00		
STRO	WHR	Additive genetic effects	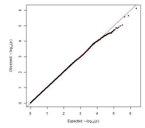	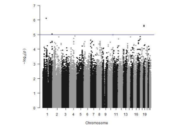	1.0225E+00	1.0023E+00	1.0080E+00	Phase III
		GxE interaction effects	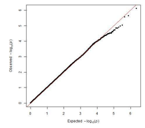	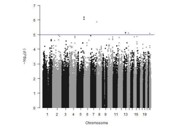	1.0368E+00	1.0037E+00		
BMI	PC1	Additive genetic effects	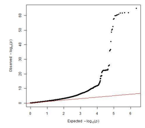	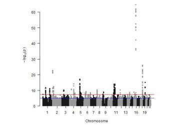	1.3639E+00	1.0016E+00	1.0549E+00	Phase II
		GxE interaction effects	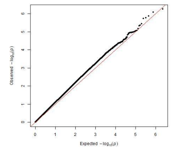	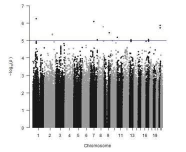	1.1342E+00	1.0006E+00		
WC	PC1	Additive genetic effects	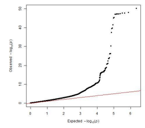	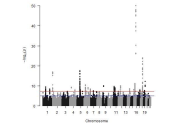	1.3174E+00	1.0014E+00	1.0619E+00	Phase II
		GxE interaction effects	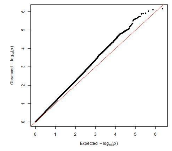	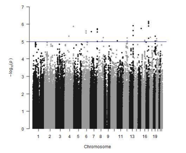	1.1429E+00	1.0006E+00		
WHR	PC1	Additive genetic effects	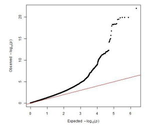	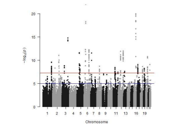	1.2731E+00	1.0012E+00	1.0214E+00	Phase II
		GxE interaction effects	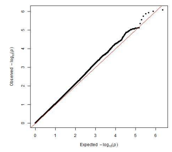	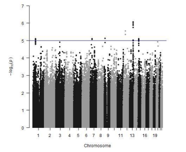	1.0502E+00	1.0002E+00		
BF	PC1	Additive genetic effects	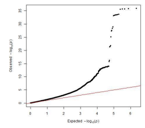	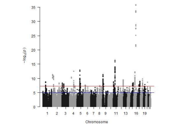	1.3631E+00	1.0016E+00	1.0187E+00	Phase II
		GxE interaction effects	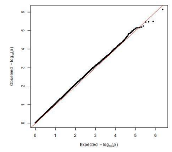	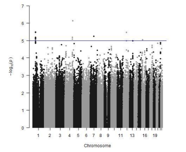	1.0590E+00	1.0003E+00		

References

[1] Emelia J Benjamin, Michael J Blaha, Stephanie E Chiuve, Mary Cushman, Sandeep R Das, Rajat Deo, Sarah D De Ferranti, James Floyd, Myriam Fornage, Cathleen Gillespie, et al. Heart disease and stroke statistics—2017 update: a report from the american heart association. *circulation*, 135(10):e146–e603, 2017.

[2] Nigussie Assefa Kassaw, Ang Zhou, Anwar Mulugeta, Sang Hong Lee, Stephen Burgess, and Elina Hyppönen. Alcohol consumption and the risk of all-cause and cause-specific mortality—a linear and nonlinear mendelian randomization study. *International Journal of Epidemiology*, 53(2):dyae046, 03 2024.

[3] RE Ferner and Jacky Chambers. Alcohol intake: measure for measure. *BMJ (Clinical research ed.)*, 323(7327):1439–1440, 2001.

[4] Muktar Ahmed, Ville-Petteri Mäkinen, Anwar Mulugeta, Jisu Shin, Terry Boyle, Elina Hyppönen, and Sang Hong Lee. Considering hormone-sensitive cancers as a single disease in the uk biobank reveals shared aetiology. *Communications Biology*, 5(1):614, 2022.

[5] Aysu Okbay, Jonathan P Beauchamp, Mark Alan Fontana, James J Lee, Tune H Pers, Cornelius A Rietveld, Patrick Turley, Guo-Bo Chen, Valur Emilsson, S Fleur W Meddens, et al. Genome-wide association study identifies 74 loci associated with educational attainment. *Nature*, 533(7604):539–542, 2016.
